# Manipulation of Behavioral Decline in *Caenorhabditis elegans* with the Rag GTPase *raga-1*

**DOI:** 10.1371/journal.pgen.1000972

**Published:** 2010-05-27

**Authors:** Matthew A. Schreiber, Jonathan T. Pierce-Shimomura, Stefan Chan, Dianne Parry, Steven L. McIntire

**Affiliations:** Ernest Gallo Clinic and Research Center, Department of Neurology, University of California San Francisco, Emeryville, California, United States of America; Stanford University Medical Center, United States of America

## Abstract

Normal aging leads to an inexorable decline in motor performance, contributing to medical morbidity and decreased quality of life. While much has been discovered about genetic determinants of lifespan, less is known about modifiers of age-related behavioral decline and whether new gene targets may be found which extend vigorous activity, with or without extending lifespan. Using *Caenorhabditis elegans*, we have developed a model of declining neuromuscular function and conducted a screen for increased behavioral activity in aged animals. In this model, behavioral function suffers from profound reductions in locomotory frequency, but coordination is strikingly preserved until very old age. By screening for enhancers of locomotion at advanced ages we identified the ras-related Rag GTPase *raga-1* as a novel modifier of behavioral aging. *raga-1* loss of function mutants showed vigorous swimming late in life. Genetic manipulations revealed that a gain of function *raga-1* curtailed behavioral vitality and shortened lifespan, while a dominant negative *raga-1* lengthened lifespan. Dietary restriction results indicated that a *raga-1* mutant is relatively protected from the life-shortening effects of highly concentrated food, while RNAi experiments suggested that *raga-1* acts in the highly conserved target of rapamycin (TOR) pathway in *C. elegans*. Rag GTPases were recently shown to mediate nutrient-dependent activation of TOR. This is the first demonstration of their dramatic effects on behavior and aging. This work indicates that novel modulators of behavioral function can be identified in screens, with implications for future study of the clinical amelioration of age-related decline.

## Author Summary

As humans and animals age, there is an inevitable decrease in functional capacity. Elderly individuals can suffer from a decline in motor function, or the ability to move. Genetic studies in model organisms have led to the identification of genes that can prolong lifespan. Elongation of lifespan is less appealing, however, if there is not also an extension of vitality or enhanced functionality. Here, we have used a genetic model organism, the nematode worm *Caenorhabditis elegans*, to screen for mutations that result in enhanced vitality in older animals. We identified a new modifier of the aging of motor function, RAGA-1, a protein present in species from worms and fruit flies to humans. Animals with a *raga-1* mutation move more vigorously at advanced ages and also live longer, on average, than wild-type. In contrast, animals engineered with an excessively active version of RAGA-1 show decreases in behavioral activity earlier in life than wild-type and a strikingly shortened lifespan. This offers the possibility that manipulating *raga-1* could also produce beneficial effects, such as enhanced vitality, in aging humans.

## Introduction

The decline of motor function with age can lead to personal decreases in quality of life and medical morbidity, and also poses challenges for society as a whole. Life expectancy has increased substantially and may be expected to increase even further as a result of ongoing research into the molecular underpinnings of aging [1]. It will be increasingly urgent to identify ways to slow declining neuromuscular function. While research with model organisms has identified a large number of genes influencing lifespan itself across diverse species [2]–[10], much less is known about modifiers of the age-related decline in behavioral function.

Like other animals, *Caenorhabditis elegans* exhibits behavioral decline over the lifespan [11], [12]–[14]. Previously, genes which increase lifespan when reduced in function (in particular *eat-2*, *daf-2*, and *age-1*) have also been shown to slow motor decline, using locomotory speed as an index [12], [13], [15], [16]. Here we ask whether novel genes could be identified which improve behavioral aging by using a screen for enhanced locomotion in old animals, directly targeting activity as an endpoint. From this screen, we have identified *raga-1* as a novel genetic target that alters the rate of behavioral aging.

*raga-1* is the sole worm orthologue of the evolutionarily conserved ras-related GTPase RagA. RagA proteins include *S. cerevisiae* Gtr1p [17], *Drosophila* RagA (dRagA, CG11968) [18] and mammalian RagA and RagB [19], [20]. *raga-1* is a particularly intriguing aging modulator, as it was recently identified as an upstream, amino acid-sensitive activator of the target of rapamycin complex 1 (TORC1) pathway [18], [21]. This pathway has been strongly implicated in regulating both growth and aging properties in many organisms: reducing TORC1 pathway activity extends lifespan in organisms from yeast to flies, including *C. elegans* [22]–[27]. Mammalian lifespan also responds to decreased TORC1 activity: in a recent study, rapamycin-treated mice were found to live longer even with treatment in mid-life [28]. This suggests that the TORC1 pathway's role in modulating lifespan is conserved. Here we show that genetic manipulation of *raga-1* in this pathway can have positive or negative effects on the behavioral vitality and lifespan of the whole organism. Our results with *raga-1* suggest we have identified a “tunable” upstream modulator of TORC1 capable of altering behavioral aging and lifespan. Upstream of TORC1, *raga-1* may be a potential target for new interventions into the process of behavioral aging.

In this work we have sought to develop a new model of *C. elegans* age-related motor decline by examining both the speed and coordination of swimming, using digital analysis to provide quantitative information about behavioral aging. We have conducted a novel screen based on the hypothesis that new aging-related genes could be identified which prolong behavioral vitality, with or without also extending lifespan. We have identified *raga-1* as one such new modulator of behavioral aging in *C. elegans*.

## Results

### Development of a *C. elegans* swimming model of behavioral aging

Previous studies have described behavioral decline in aging *C. elegans* using direct observation of locomotion as the animals crawled on solid media [12]–[14] or swam in liquid [15]. These studies have provided useful baseline information on the aging of behavior, but have relied on observer determinations of velocity or qualitative assessment of locomotion to chart decline. More recently, digital analysis was used to show an age-dependent exponential velocity decline in nematode crawling on solid media [16]. Here we develop a new model of *C. elegans* locomotory aging with a study of worm swimming using video analysis tools [29]. Worm swimming offers an alternative to crawling as a neuromuscular activity to monitor [15], [30] with several useful features: immersing worms in liquid is sufficient to initiate swimming, which helps standardize conditions and enhances the feasibility of genetic screens; a large amount of data can be gathered quickly as swimming cycles are rapid; and there is increased freedom of movement compared with crawling on solid media, so abnormal movements, if present, may be easier to detect.

**Figure 1:**
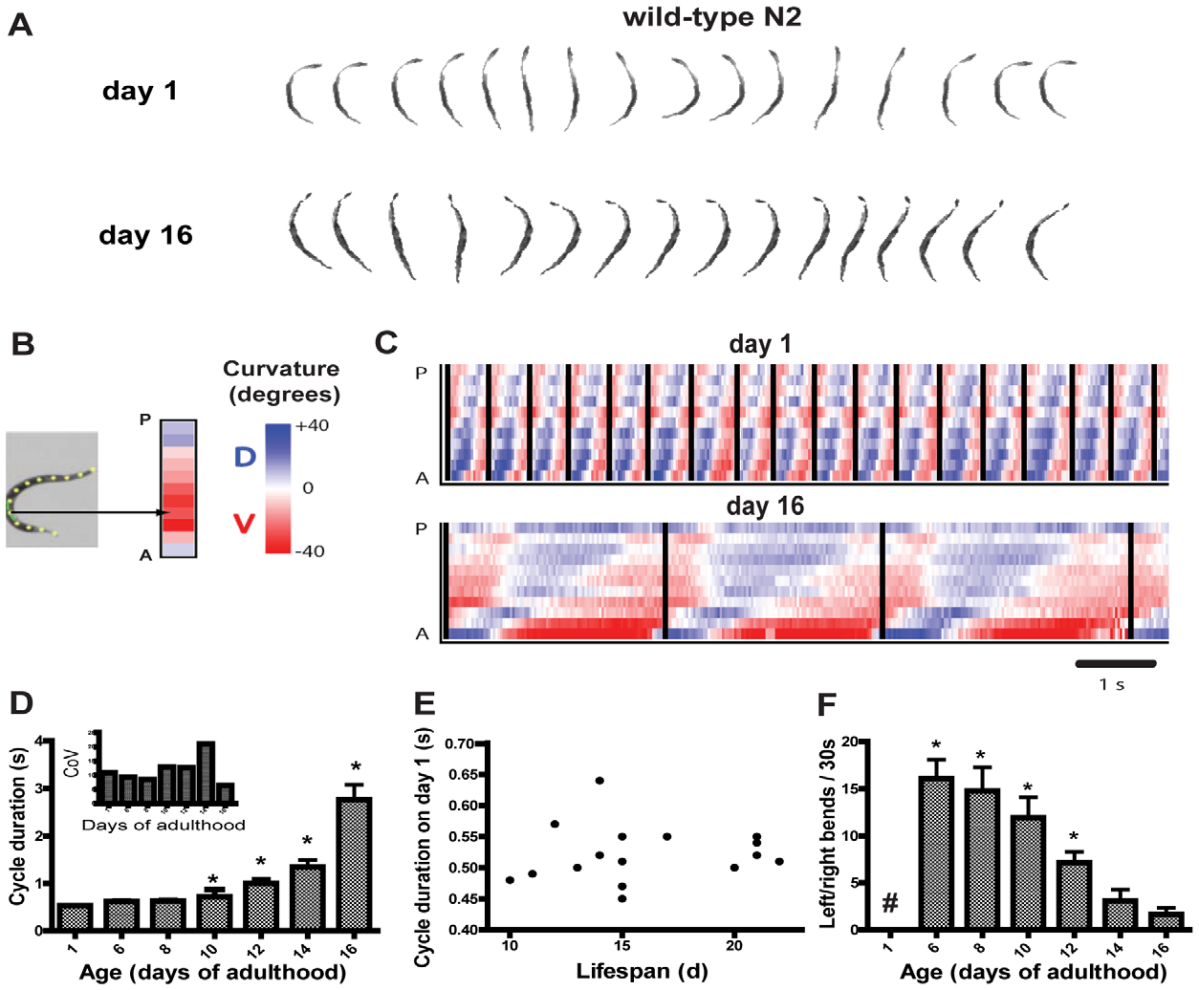
Longitudinal study of swimming over the lifespan of wild-type N2 worms. (A) Collage of swimming postures from one complete swim cycle. Every frame, or every seventh frame, is shown from a day 1 and day 16 animal respectively, from 30 frames per second movies. (B) Building of curvature matrix from a spine along the worm midline. Each angle along the worm is depicted using scaled blue or red coloring for curvature in the dorsal/ventral plane. The anterior is represented at the bottom of the matrix. (C) Representative curvature matrices from the same adult on day 1 of adulthood (top) and day 16 (bottom) show dramatic slowing of the swim cycle with relative preservation of waveform. (D) Mean (+/−S.D.) swim cycle duration in seconds (n = 18, 17, 16, 14, 12, 10, and 6 for days 1, 6, 8, 10, 12, 14, and 16 respectively). The increase is curvilinear, with rapid increases following a period of relative stability earlier in adulthood (*p<.05 versus day 1; Dunn's test). (Inset) Coefficient of variation for swim cycle duration on each day tested. Variability of cycle duration is fairly stable over the lifespan after correction for increases in the mean. (E) Plot of mean cycle duration on day 1 of adulthood versus lifespan for each individual in the cohort showing no correlation or predictive value of the cycle duration on day 1. Similar plots from later ages were not predictive of lifespan either (data not shown). Two worms' natural lifespan durations were not determined (i.e., were censored) due to accidental death. (F) Age-dependent incidence of left/right bends, out of the usual dorsal/ventral swimming plane (mean+/−S.E.M; *p<.05 versus day 1, Dunn's test; #, none were observed on day 1). For this experimental cohort, the mean lifespan was 16.0+/−0.9 days (mean+/−S.E.M.; n = 16 deaths observed).

In worm swimming, waves of curvature propagate along the body from anterior to posterior. In series of still frames, these appear as oscillations from “C”-shaped to a nearly straight posture and then to a reverse “C”-shape and back again, completing one cycle or “swim stroke” (Figure 1A). These can be examined to identify changes in the timing and coordinated postures, or kinematics, of swimming. Previously we used video image analysis to characterize swimming of young adults [29]. Here we examined aging nematodes in a longitudinal study. In this analysis, movies were taken of individual animals at time points over the worm's lifespan. One cycle for a young adult on day 1 (sequential frames are displayed), and the same adult at day 16 (every seventh frame is displayed), are shown in Figure 1A, which illustrates the similarity in swimming postures over the lifespan. Stills were image processed to convert each worm into a 13-point spine along the midline. To help visualize and to quantitate these postures, each of the 11 angles formed by the sets of three consecutive points along the worm spine were converted to color (red or blue) and then used to build a curvature matrix by arraying the spine data associated with each frame together sequentially to form a time series (Figure 1B and 1C). In these curvature matrices, each forward swim cycle begins at the moment the head initiates a bend to the ventral side. Cycles are marked by vertical bars in Figure 1C.

Data from a cohort of aging animals showed that swimming slowed profoundly over the lifespan (Figure 1D; Video S1, S2). The amount of slowing of the behavior is non-linear, with peak performance in youth, a substantial period of preserved function in “middle age” followed by a rapid functional decline (increase in cycle duration) in older worms, beginning around day 10–12 of adulthood (mean lifespan in this cohort was 16 days). These dynamics of changing performance contrast with crawling velocity which also slows with age, but very closely follows an exponential decay starting early in life [16]. In addition to an increase in cycle duration, the cycles become less uniform as animals age; cycle length is very strongly rhythmic and uniform on day 1, but later culminates in longer and more variable cycle durations in old age. (However, these increases in variability correspond fairly closely with the increased mean duration, as the coefficient of variation indicates; Figure 1D, inset). The predictive value of swim cycle duration was also examined, since biomarkers of aging are very useful [16], [31]. As previously shown for crawling velocity [16], the swimming cycle duration of young adults, for example on the first day of adulthood, was not predictive of the lifespan of the individual (Figure 1E; swim cycle durations on other days of life were also not predictive of lifespan, data not shown). Also, while the rate of decay of crawling velocity starts early in life and fits a single exponential curve which can be used to predict lifespan [16], we have not yet found early lifespan swimming performance parameters that are similarly predictive. These differing dynamics of decay suggest that the causes of swimming and crawling performance decline may differ to some extent.

In contrast, the coordination (or kinematics) of the behavior changed relatively little, as seen in movie stills (Figure 1A) and in curvature matrices (Figure 1C). As suggested by the similarity of the stills from the young and old animals' cycles, the postures adopted by the worm in swimming remained similar across the lifespan, but the rate of change between postures was greatly lengthened in older animals. This suggests that neuromuscular coordination persists well into old age in worms. However, though the swim cycles remained similar in form, we also noted an age-dependent increase in bends out of the usual dorsal/ventral swimming plane. In these bending movements, swimming cycles were interrupted by rolling or head-lifting movements to the left or right (up or down relative to the observer), then a return to normal swim cycle behavior (Video S3). These were noted extremely infrequently during our observation of young, wild-type animals on the first day of adulthood, but by day 6 of adulthood were readily seen, then decreased in number with age as worms moved more slowly (Figure 1F).

### Identification of *raga-1* as a modifier of behavioral aging

We hypothesized that novel genetic modifiers of the rate of behavioral aging could be found by directly screening for enhanced behavioral vitality in old age. Using the endpoint of increased activity in old age, we performed an RNAi pilot screen to identify such novel mediators of age-related behavioral decline. Early in the course of this pilot we found that *raga-1* RNAi treatment improved locomotion at advanced ages when compared with empty vector control (unpublished observations). We were attracted to *raga-1* as a target given its recent implication in mediating a link between nutrient sensing and activation of TORC1 [18], [21], especially since TOR itself has a role in aging in *C. elegans* [23], [27], [32]–[35] and yeast and flies [22], [25], [36]. As an initial step, we confirmed qualitatively that a deletion mutant, *raga-1(ok386)*, showed similar alterations in age-related decline as the RNAi knockdown of *raga-1* (data not shown).

**Figure 2:**
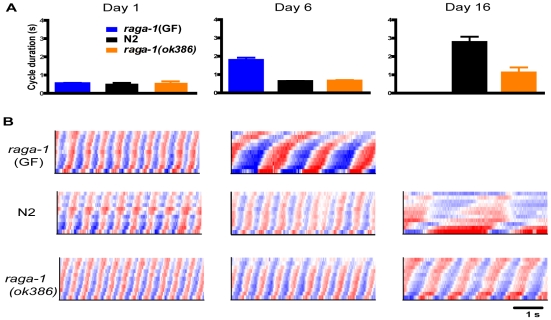
Comparison of swimming in aging *raga-1(ok386)*, transgenic *raga-1*(GF), and wild-type animals. (A) Comparison of mean (+/−S.E.M.) cycle duration for *raga-1(ok386)*, *raga-1*(GF) and wild-type N2. (Left) Mean cycle duration on day 1. Note the cycle lengths are similar for vigorous day 1 adults of all three genotypes. (Middle) Cycle durations on day 6 of adulthood; *raga-1*(GF) show substantial increases compared with N2 and *raga-1(ok386)* (*, p<.05, Dunn's test) (Right) Cycle durations on day 16, showing lengthening of wild-type cycles while *raga-1 (ok386)* still exhibits comparatively short cycles, though also increased from day 6. (B) Representative curvature matrices on day 1 (left column), day 6 (middle) and day 16 (right) for *raga-1*(GF) (top row), wild-type (middle) and *raga-1(ok386)* (bottom). As early as day 6, *raga-1*(GF) worms show slowing cycles; bends are also deeper than wild-type, suggesting qualitative changes in swimming occurred as well. By day 16, *raga-1(ok386)* worms swim substantially faster than wild-type (*, p<.05, Mann-Whitney U).

We compared *raga-1(ok386)* swimming with wild-type over the lifespan. Both wild-type and *raga-1(ok386)* worms showed vigorous, rapid, and highly rhythmic swimming on day 1 of adulthood (Figure 2; Video S1, S4). These findings correspond well with previous studies of young animals and behavioral aging in worms [13], [15], [16]. The similar swimming form, short cycle duration, and low variability all suggest that *raga-1* mutant worms developed healthy coordination for swimming and are, from the standpoint of locomotion, vigorous young adults much like wild-type worms. We then examined *raga-1(ok386)* worms later in life. As expected from the preliminary RNAi result with *raga-1*, over the lifespan wild-type and mutant swimming behavior diverged. At an advanced age, on average *raga-1(ok386)* mutant worms exhibited more rapid swimming than wild-type, as seen in comparing curvature matrices from an example individual of each genotype (Figure 2B; Video S2, S5). Despite differing swim rates, the waveforms of both appeared similar, suggesting that the underlying aging process of swimming is not affected greatly by the mutation. The divergence in behavioral aging corresponds to a shorter mean cycle duration in older mutant *raga-1(ok386)* worms (Figure 2A). However, ultimately *raga-1(ok386)* worms stop swimming and undergo a terminal phase of life with no coordinated locomotion as do wild-type worms. Finally, *raga-1(ok386)* worms also exhibited fewer of the age-dependent left/right, out of plane bends than wild-type worms as they swam (Figure S1). Overall, these results indicate that rates of behavioral decline are altered in *raga-1(ok386).*

### Genetic alterations of *raga-1* affect lifespan

**Figure 3:**
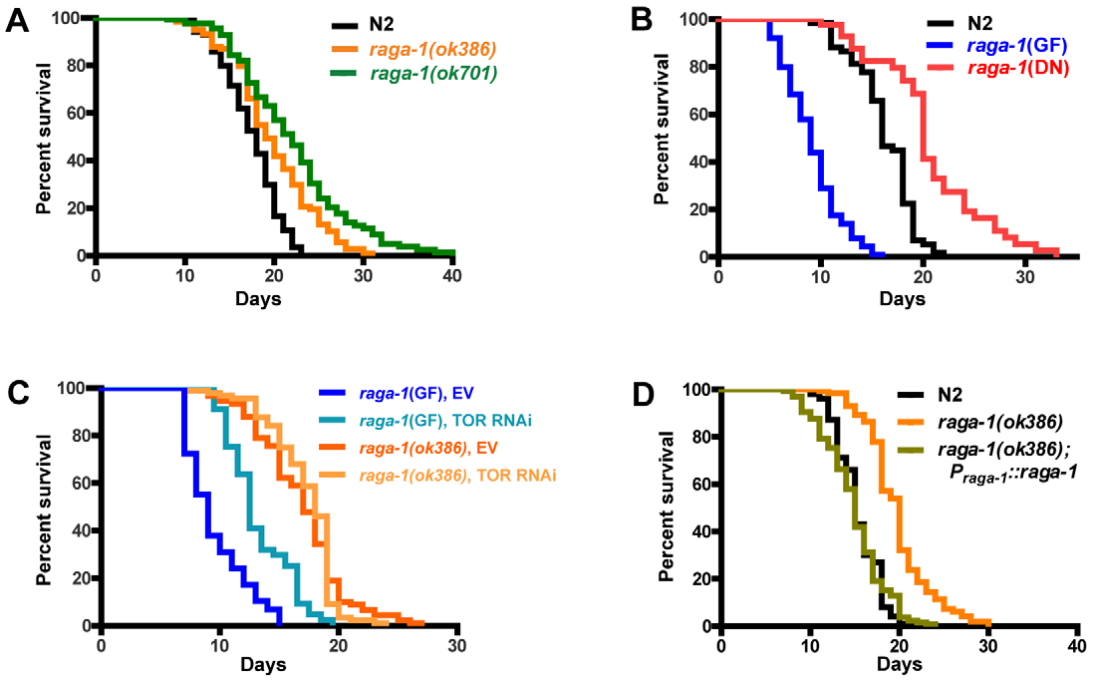
Genetic alteration of *raga-1* affects lifespan. Lifespan assay results from representative experiments (see Table S1 for quantitative data and results of all experiments). (A) Comparison of *raga-1(ok386)* and *raga-1(ok701)* with wild-type N2 survival at 20°C. Strains harboring alleles *ok386* and *ok701* consistently showed increases in lifespan (N = 7 and 4 trials, respectively). (B) Transgenic expression of *raga-1* dominant negative (DN) increased, while *raga-1* gain of function (GF) decreased, lifespan versus wild-type (N = 5 and 4, respectively). (C) Effect of CeTOR (*let-363*) RNAi knockdown during adulthood on lifespan of *raga-1*(GF) and *raga-1(ok386)* at 25°C. See Table S3 for quantitative results of RNAi experiments. (D) Transgenic extrachromosomal expression of wild-type *raga-1* under its native promoter rescues lifespan to wild-type duration in the *raga-1(ok386)* background.

Given that several important lifespan-influencing genes have been shown to slow the rate of behavioral aging [12], [13], [15], [16], we investigated whether overall lifespan was also affected by *raga-1* mutations. *raga-1* deletion mutant alleles (Figure S2) available from knock-out consortia were tested in survival analyses (Figure 3A; Table S1). *raga-1(ok386)* is predicted to preserve 48 N-terminal amino acids from RAGA-1, resulting in a deletion product comprised of the N-terminal 15% of the protein. *raga-1(ok386)* showed increased lifespan across multiple survival analyses (18.5+/−3.6% increase in lifespan; mean+/−S.E.M.; n = 7). A second *raga-1* deletion allele, *raga-1(ok701)*, which is predicted to preserve a much larger N-terminal fragment of 158 amino acids (plus 4 non-native amino acids coded by sequence 3′ to end of the deletion), or approximately 50% of the total protein length, produced a stronger lifespan extension (25.5+/−7.3%; n = 4), but also showed more severe pleiotropic phenotypes, for example reduced brood size (Figure S3; Table S2). A third deletion allele, *raga-1(tm1862)*, which is predicted to lead to a fusion of 27 N-terminal RAGA-1 amino acids and 39 non-RAGA-1 amino acids after a frameshift introduced by the deletion, did not exhibit lifespan longer than wild-type (N2) (Table S1).

To further explore the effects of altering *raga-1* function in worms, we designed genetic modifications of *raga-1* based on studies of RagA function in mammalian cells and *Drosophila* [18], [21]. These studies made use of site-directed mutation of specific residues highly conserved among ras family proteins to produce dominant negative (DN) and gain of function (GF) forms of Rag proteins due to constitutive GDP- and GTP- binding, respectively. Previously published results indicated that an orthologous Rag GF was sufficient to induce activation through this pathway and resulted in increased TORC1 pathway activity [21]. In addition, transgenic expression of Rag GF and DN constructs in *Drosophila* cells resulted in increased and decreased size of the cells respectively, suggesting manipulation of downstream cellular TORC1 activity led to changes in cellular growth properties [18]. In order to examine these mutations' effects on the animal as a whole, we made corresponding DN (T18N in *C. elegans raga-1*) and GF (Q63L) mutations in worm *raga-1* and expressed these transgenically in the wild-type N2 background using the *raga-1*operon promoter (Figure S2B).

*raga-1*(DN) expression closely phenocopied *raga-1* deletion mutants *ok386* and *ok701*, resulting in an increase in lifespan similar to *raga-1(ok386)* (17.7+/−3.5% (n = 5); Figure 3B). In addition, similar to these mutants, *raga-1*(DN) transgenic worms showed cold-dependent reductions in brood sizes at 14C (Figure S3, Table S2; this appeared to be due to cold-dependent increases in embryonic lethality, data not shown). This is reminiscent of the cold-sensitivity of orthologous yeast gtr1 mutants, and may suggest evolutionarily conserved functions which are disrupted at lower temperatures [17]. The DN phenotypic similarity to the deletion mutants suggests expression from the *raga-1* operon promoter functionally approximates the native expression pattern.

Next we examined the effects of expressing a transgenic *raga-1* gain of function mutation under the control of the *raga-1* promoter. When expressed in a wild-type background, *raga-1*(GF) produced pronounced phenotypic changes, most notably a substantially decreased life span (Figure 3B). Reductions of lifespan can be difficult to interpret, in particular because worms may be generally sickened by genetic manipulations. However, the lack of general debility of *raga-1*(GF) is suggested by the absence of visible developmental anomalies and the relative preservation (though incomplete) of brood size (Figure S3, Table S2). In addition, expression of wild-type *raga-1* under its own promoter in the *raga-1(ok386)* background rescued lifespan to N2 lengths (Figure 3), as well as partially rescued the increase in swimming cycle duration with aging (Figure S4). This suggests that changes in lifespan with different *raga-1* transgenic versions were due to the specific properties of the transgenic version of RAGA-1 expressed, and that expression of *raga-1* under this promoter sufficiently mimics native expression to rescue lifespan effects of the mutant. Possible explanations for the incomplete rescue of the swimming phenotype may be the relatively small sample size relative to lifespan experiments leading to higher variance in the result, or a higher sensitivity of this phenotype to the exact levels of RAGA-1 being expressed.

We then examined the effects of *raga-1*(GF) on behavioral aging. At young ages, these worms moved vigorously, also suggesting that these animals were not generally sick (Figure 2B; Video S6). However, swimming of *raga-1*(GF) changed rapidly with age, corresponding with its shortened lifespan (Figure 2B). Pooled data on swim cycle duration revealed an increase between day 1 and day 6 greater than that observed for wild-type or *raga-1(ok386)* (Figure 2A; Video S7). However, along with changes in swimming cycle duration, additional alterations in *raga-1*(GF) swimming were present and especially notable in day 6 worms, which bend more strongly than wild-type worms of any age (note the darker shades of red and blue indicating more pronounced postural curvature). Crawling on plates is also “loopy” on day 1 suggesting developmental changes in *raga-1*(GF) locomotion (data not shown). Thus, the effects of the GF are not necessarily restricted to modification of the aging process but also possibly affect development. The properties of the *raga-1*(GF) are likely to be a composite of accelerated aging and other changes due to the activity of the transgene during development. The results with *raga-1*(GF) worms are opposite to the effects of the dominant negative transgene on lifespan, suggesting that changing the activation of the TORC1 pathway by decreasing or increasing *raga-1* activity can alter both lifespan and behavioral aging in either a positive or negative direction, respectively.

Since RagA activates the TORC1 pathway [18], [21], we tested the effects of CeTOR knockdown in different *raga-1* backgrounds by TOR RNAi (Figure 3C, Table S3). While *raga-1*(GF) exhibited increased lifespan with TOR RNAi treatment compared to empty vector, *raga-1(ok386)* did not. This suggests that, as in other systems, *raga-1* is acting through TOR to exert its effects on lifespan. Application of TOR RNAi earlier in the lifespan (feeding from larval stages onward) produced developmental alterations or arrest, as previously shown with both RNAi and with CeTOR mutations [33], [37]; presumptive loss of function CeTOR mutations are not viable as homozygotes (CeTOR is also known as *lethal* (*let-*) *363*) [37]. Altering *raga-1* activity may produce much less severe changes in phenotype than direct alteration of TOR itself (which also participates in a second complex, TORC2 [38], [39]) by producing milder changes in TORC1 activity than would be caused by direct manipulation of CeTOR itself.

We examined the effects of an *rsks-1* (ribosomal S6 kinase) mutant on lifespan and swimming to investigate the effects of an additional genetic alteration in the TORC1 pathway. Ribosomal S6 kinase is a downstream mediator of TORC1 function in many systems [24], [40], [41], [42]. As previously shown [26], [35], [43], *rsks-1*mutation produced a moderate increase in lifespan compared to wild-type (Figure S7). In double mutants, *raga-1(ok386)* further increased *rsks-1(ok1255)* lifespan, suggesting that these genes may act at least partially in different pathways to exert lifespan effects. Thus, *raga-1* likely acts through other TORC1 effectors, at least under these experimental conditions. We also examined the swimming behavior of this additional TORC1 pathway mutant (Figure S4). On day 1 of adulthood, *rsks-1(ok1255)* animals showed similar swim cycles and cycle durations to wild-type. Older day 16 *rsks-1(ok1255)* animals showed significantly less increase in swim cycle duration than wild-type, similar to *raga-1(ok386)*. However, the swim cycles changed in form and were interrupted frequently by uncoordinated movements, for example twisting movements out of the plane of swimming and kinking or curling. Changes in swimming pattern can be seen in the color pattern of swim cycles reflecting alterations in the angles adopted by the animal during swimming; for example, in Figure S4, panel E the tail remains red due to a lack of movement, whereas in ordinary swim cycles the tail oscillates between red and blue reflecting movement to either side. Qualitatively, these animals appeared uncoordinated as they aged both when examined on plates or in liquid. This suggests that while genetic alterations interfering with the TORC1 pathway may share the property of improving swim cycle duration at advanced ages, they may also have pleiotropic effects on other aspects of locomotion. Although the mechanisms may be molecularly distinct, this combination of uncoordinated locomotion and long lifespan is reminiscent of *daf-2(e1370)* (a Class II *daf-2* allele with pleiotropic phenotypes, including being uncoordinated (unc) [44]), and highlights the point that not all mutants with increased longevity will have improved locomotion in old age.

### *raga-1* animal size and expression pattern

**Figure 4:**
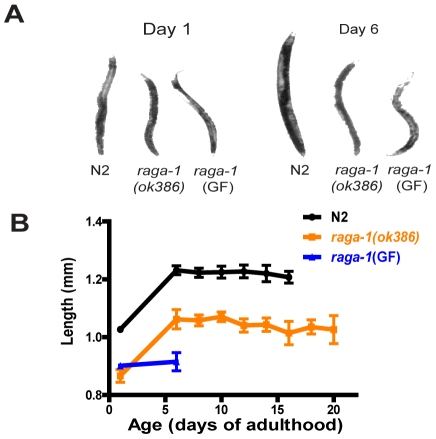
Size of adult animals over the lifespan for wild-type N2, *raga-1(ok386)*, and *raga-1*(GF). (A) Representative images of animals of each genotype to illustrate size differences. (B) Sizes of adult animals derived from measurement of length in video stills used for swimming analysis. Note differences in wild-type and *raga-1(ok386)* by day 1 of adulthood, suggesting different larval growth properties, which persist into adulthood even though animals continue to increase in size as adults. *raga-1*(GF) animals do not show any adult increase in size by day 6.

Since TORC1, and Rag proteins more specifically, modulate growth properties, we examined adult animal size (except for the germ line, adults are post-mitotic in *C. elegans* but still increase in size with age, especially during early adulthood [45], [46]). On day one *raga-1(ok386)* adults were smaller than wild-type (Figure 4). This is reminiscent of expression of a dominant negative dRagA in *Drosophila* cells, which resulted in decreased size of the transgenic cells, and may be due to downregulation of TORC1 activity in a shift to “low growth” conditions [18]. The adult size increases in wild-type and *raga-1(ok386)* were parallel, suggesting that their rate of growth was similar in adulthood, and that the size differences as adults were due to changes during development. Surprisingly, *raga-1*(GF) expression also resulted in smaller animals (Figure 4). While *raga-1(ok386)* and wild-type animals continued to increase in size from day 1 to day 6 of adulthood, *raga-1*(GF) animals showed little increase in size over this period.

To investigate the tissue basis of RAGA-1 action, we examined the expression pattern of the *raga-1*operon promoter driving mCherry or GFP in a wild-type background. Expression was widespread, perhaps ubiquitous, in larval animals (data not shown), similar to CeTOR [37]. In adults, expression was more restricted, and was most notable in the gut, unidentified head and tail neurons, and the distal tip cell of the somatic gonad (Figure S5). Examination of an additional independently generated line showed a similar pattern, but with higher adult levels of hypodermal expression (especially in the head), and in the entire somatic gonad including the spermatheca and gonadal sheath cells (data not shown). This restricted pattern of expression raises a question about the role of RAGA-1 in specific tissues in terms of behavior and aging in adulthood. To address this, we carried out experiments designed to test for tissue-specific phenocopy of the dominant negative or gain of function effects on lifespan. We generated transgenic lines using previously characterized promoters to drive expression of gain of function or dominant negative *raga-1* in neurons, muscle, gut, or the distal tip cell of the somatic gonad. However, none of these lines showed substantial effects on lifespan (Table S4), which suggests larval, ubiquitous expression may be key to the RAGA-1 effect on lifespan. RNAi experiments also support this possibility, as *raga-1* RNAi treatment in adulthood only did not extend lifespan, whereas RNAi treatment from hatching did prolong lifespan (Figure S6; Table S3). Alternatively, expression in the adult may also play a role but this may be dependent on tissues (or combinations of tissues) not tested here.

### *raga-1* genetically interacts with other aging genes

We combined *raga-1(ok386)* with other mutations known to affect lifespan (Figure S7; Table S5), focusing on those which mediate the complex relationship of metabolic and dietary status and lifespan. Results with *daf-2*/*daf-16* insulin/insulin-like growth factor (IIS) pathway mutants suggest that *raga-1* lifespan extension is dependent on *daf-16*, as *daf-16*; *raga-1* double mutants show shortened lifespan similar to *daf-16* alone. Upstream in the same pathway, results with *daf-2(e1368)* (a phenotypically less severe, Class I allele of *daf-2* [44]) indicated that *raga-1(ok386)* lifespan was extended even further in combination with *e1368*. Examination of the stronger, Class II allele *daf-2 (e1370)* was more difficult to interpret because the strain appeared very debilitated with many morphological abnormalities accumulating over time, for example extrusion of the gut through the vulva. This may account for the biphasic appearance of the survival curve (Figure S7). However, maximum lifespan of *raga-1 (ok386)*;*daf-2(e1370)* double mutants was similar to *daf-2(e1370)* alone. Taken together, the results suggest that *raga-1* and the *daf-2* pathways interact, and one possibility is that *raga-1* may be upstream of this pathway such that *daf-16* function is needed to at least partially carry out the beneficial effects of *raga-1* mutation on lifespan extension. Previously it has been shown that the TOR pathway and *daf-2* pathway interact in complex ways [23], [34], [35], and this data suggests these interactions may occur at many levels of the pathway.

We also examined the effects of two transcription factors which are crucial for dietary restriction-mediated longevity increases, *skn-1* and *pha-4* [34], [47], [48]. Under *pha-4* RNAi treatment, *raga-1(ok386)* lifespan was only slightly longer than N2 (Figure S7), suggesting *pha-4* does not strongly contribute to *raga-1(ok386)*'s extended lifespan, at least under these conditions. In contrast, a *skn-1* mutation reversed the lifespan extension of *raga-1(ok386)* (Figure S7). This is consistent with previous results showing that *skn-1* also interacts with the IIS pathway [48], and suggests that one effect of *raga-1* mutation is to reduce signaling through the same output mechanisms as IIS, either acting upstream or in parallel with *daf-2*.

**Figure 5:**
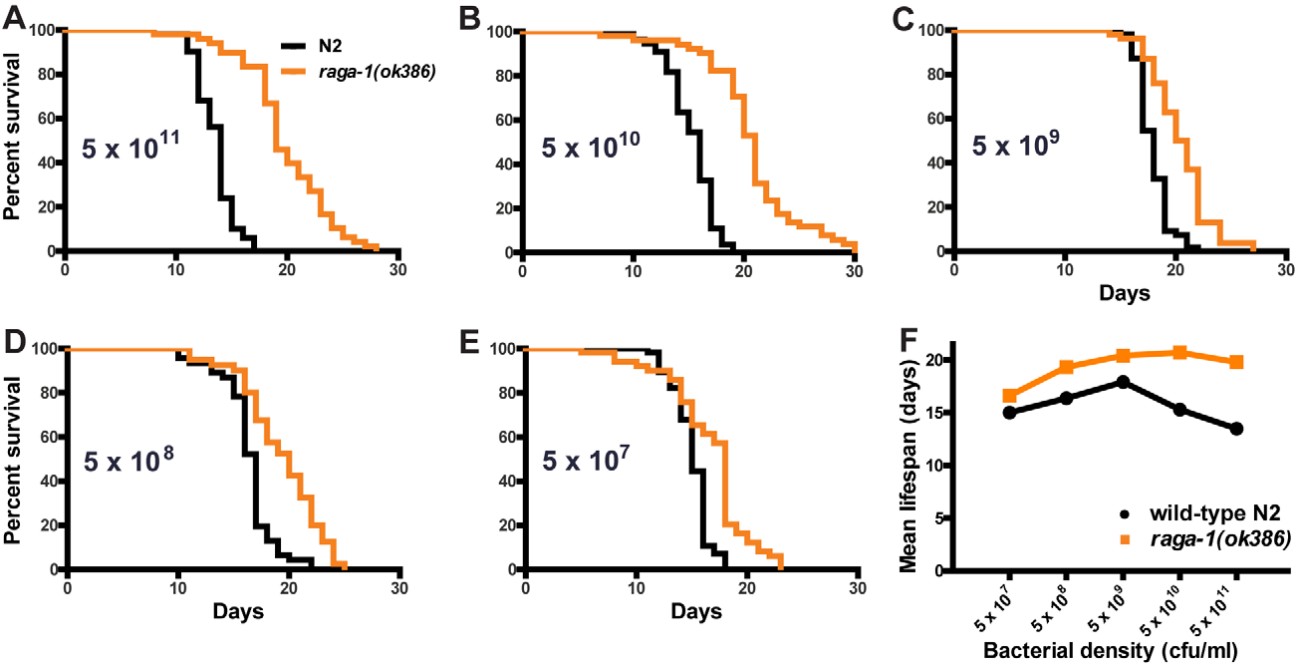
Effect of dietary restriction on *raga-1(ok386)* lifespan. (A–E) Survival curves at 25°C under varying dietary conditions. The bacterial density of the food source is shown on each panel. The largest increase in lifespan seen for *raga-1(ok386)* versus N2 wild-type control is at the highest concentration of bacterial food. (F) Mean (+/−S.E.M.) lifespan plotted versus bacterial density. Wild-type N2 shows the expected parabolic response to dietary restriction, while *raga-1(ok386)* is much less sensitive.

The requirement of *skn-1* function, as well as the role the TORC1 pathway plays in nutrient sensing, suggested that the role of RAGA-1 in lifespan may vary with dietary conditions. Double mutants with *eat-2* alleles *ad1116* and *ad465* indicated that *raga-1(ok386)* was not susceptible to the lifespan extension effects of dietary restriction using this genetic model, as the double mutants had similar lifespan extension to the respective single *eat-2* mutants (Figure S8; Table S5). To look in more detail at this possibility, we tested the effects of dietary restriction directly by varying the concentration of bacterial food [49]. In these experiments, *raga-1(ok386)* responses to dietary restriction were strongly blunted in comparison to wild-type N2 (Figure 5; Table S6). The strongest effect of *raga-1(ok386)* on lifespan was at the highest concentration of bacterial feeding. This suggests RAGA-1 may have a role in mediating the shortening of lifespan under conditions of dietary repletion or excess, so that the *raga-1(ok386)* mutation confers partial protection from the negative effects of the high concentration feeding condition on lifespan. 

## Discussion

In this study, age-dependent changes in swimming provided the basis for a screen to identify novel modulators of behavioral vitality in *C. elegans*. This screen yielded a new modulator of aging and behavior, *raga-1*. Of note, *raga-1* has not previously been identified as an aging modulator, despite multiple genome-wide RNAi screens which have identified partially overlapping groups of genes which affect lifespan [50]–[55]. Identification of *raga-1* in this screen may be due to our use of an RNAi hypersensitive strain [56], as N2 did not show lifespan extension with *raga-1* RNAi treatment, while the hypersensitive strain did show a modest, statistically significant increased lifespan (Figure S6). In addition, *raga-1* has not been identified as a downstream transcriptional target of the *daf-2*/*daf-16* pathway [57], [58]. *raga-1* does not appear to be strongly regulated by the *elt* transcriptional circuit which plays a role in longevity determination [59], and *raga-1* transcription was not revealed as strongly regulated by age [60]. This suggests that new modulators of the aging process remain to be identified.

Our characterization of swimming in aged animals provides a model for quantitating behavioral decline and monitoring the effect of genetic manipulations on this process. Previous studies have shown that canonical aging-related genes influence the rate of locomotory decline. *daf-2* pathway mutations that increase lifespan [61], [62] have been shown to improve behavioral aging; reduction of function mutants of *daf-2* showed delayed age-related decreases in locomotion [15], while downstream in the same pathway an *age-1* PI3 kinase mutant showed improved behavioral activity at advanced ages [13], [15]. *daf-2* mutations also decreased the rate of behavioral aging as measured by computer-aided examination of crawling velocity [16]. The *eat-2* mutant, a genetic model of dietary restriction [63], also modestly slowed crawling decline [16]. Here we have found remarkable preservation of coordination of swimming, as well as the profound slowing of locomotion noted previously. The cause of this slowing is unknown, but may be due to changes in muscle, especially since worm muscle shows severe decline with age, both in mass (sarcopenia) and structure, and this correlates well with qualitative assessments of crawling [14]. Additionally, as other physical properties of the worm age they may affect the mechanics of swimming, for example due to differences in cuticle stiffness. In contrast to structural changes, alterations in neuromuscular physiology might be expected to affect the coordination of swimming as well as the speed, and these may predominate during the terminal phase of decline in which there is no locomotion. This decrepit period immediately preceding death, approximately the last 15% of a worm's lifespan (unpublished observations), may correspond to the functional degradation of the nematode nervous system. In a key study of worm structural aging, however, neuronal structure was intact even in very old worms, though as noted in that study there may be functional changes in neurophysiology not readily seen at the structural level [14].

Our results identified *raga-1* as a novel, “tunable” regulator of vitality and aging in the *C. elegans* TOR pathway [25], [64]. TORC1 is a critical integrator of metabolic information, integrating signals on nutrient sufficiency, growth factor activity, and energy status to influence growth rates via regulation of downstream targets [38]. *C. elegans*' TORC1 (comprised of CeTOR and associated proteins) [23], [37] has previously been shown to influence lifespan, as CeTOR mutation and RNAi knockdown lengthen life [23], [33]. In addition, increasing autophagy and decreasing protein synthesis, processes modulated by TORC1 inhibition, have also been shown to increase worm lifespan [26], [27], [35]. Recently, *rheb-1*, an additional modulator of TORC1, was found to mediate intermittent fasting-induced lifespan increases in worms [65]; unlike *rheb-1*, *raga-1* has strong effects on behavior and lifespan under ordinary culture conditions. As these proteins interact functionally in mammalian systems [21], it will be of interest to examine their genetic interactions in *C. elegans*.

Rag proteins respond to amino acid levels and activate TORC1 when these levels are sufficient for growth [18], [21]. Speculatively, *raga-1*(GF), wild-type and *raga-1*(DN) animals may lie along of spectrum of behavioral vitality and lifespan phenotypes because of different levels of signaling through TORC1 (similarly, the *daf-2* insulin/insulin like growth factor aging pathway may also undergo tuning [66]). In this model, *raga-1*(GF) animals are “hypermetabolic,” consuming resources at an excessive rate due to inappropriate positive signaling through TORC1. Although these animals are receiving normal levels of nutrition, there may be excessive protein synthesis, and depressed levels of autophagy, leading ultimately to a shortened lifespan. These results contrast with the extension of lifespan by inhibiting translation or enhancing autophagy in worms [26], [27], [35]. *raga-1*(DN) animals may mimic these conditions of functional starvation despite the presence of adequate nutrients, due to downregulated *raga-1* signaling to TORC1. In this way, *raga-1* provides a single point of entry for manipulating the activity of the TORC1 pathway to alter vitality and lifespan in intact animals.

The interaction between nutrients and *raga-1* was also suggested by the effects of dietary restriction on lifespan. *C. elegans* have been used to model effects of dietary manipulations on lifespan extensively [47], [49], [63], [67]–[70]. Mutations in *pha-4* and *skn-1* ablate lifespan-extension by dietary restriction and result in shorter lifespan than wild-type when reduced in function. One model for the interaction of RAGA-1 and these mediators of dietary restriction is that, in *raga-1(ok386)*, the animal is in a functionally starved state, extending lifespan, and this effect is dependent on mediators of dietary restriction.

In contrast, the effects of dietary restriction on *raga-1(ok386)* closely resemble the effects of *hif-1*(hypoxia inducible factor), a downstream target of the TOR pathway in *C. elegans* [49]. As in *raga-1(ok386)*, the animals show the largest increase in lifespan under high levels of feeding (i.e., dietary repletion). These results suggest a model in which *raga-1* and the TORC1 pathway, including *hif-1*, mediate the effects of dietary repletion, perhaps shortening lifespan when food is plentiful or excessive. Reduced RAGA-1 activity may protect animals from the deleterious effects of the highly concentrated diet.

TORC1 is already an important pharmacological target in medical practice, as rapamycin is clinically used in transplant rejection prevention and oncology. Recent results show that rapamycin treatment can extend the lifespan of mice, suggesting that the effects of manipulating this pathway are evolutionarily conserved [28]. It would be desirable, however, to avoid the immunosuppressant effects of rapamycin, which are its primary clinical use. Upstream regulators of TOR may be more appealing targets because of their more restricted functions. Our findings suggest *raga-1* is a potentially useful target for upstream modulation of TOR activity, resulting in significant changes in worm locomotory function and lifespan, without as many severe effects as direct manipulation of TOR itself. *raga-1* is an attractive target for further research on interventions into the TORC1 pathway.

## Materials and Methods

### *C. elegans* strains

*C. elegans* were cultured using standard techniques [71]. Strains used were: wild-type N2 (Bristol); VC222 *raga-1(ok386)* and VC533 *raga-1(ok701)* obtained from the *C. elegans* Knockout Consortium (University of Oklahoma) via the *Caenorhabditis* Genetics Center, University of Minnesota, supported by the NIH National Center for Research Resources (NCRR); and *raga-1(tm1862)*, obtained from Prof. S. Mitani, National Bioresource Project, Tokyo Women's Medical University School of Medicine, Japan. Mutant strains were outcrossed to N2 4–6 times prior to phenotypic analysis. Additional strains used were: RB1206 *rsks-1(ok1255)*, CF1041 *daf-2(e1370)*, DR1572 *daf-2(e1368)*, CF1037 *daf-16(mu86)*, EU35 *skn-1(zu169)*, DA1116 *eat-2(ad1116)*, and DA465 *eat-2(ad465)*.

### Behavioral screen

A pilot screen yielding *raga-1* was conducted based on swimming vigor at an advanced age. RNAi hypersensitive *nre-1(hd20);lin-15b(hd126)* animals (derived from strain VH624 after outcrossing once to our N2 stock) [56] were qualitatively screened for swimming activity at advanced ages under treatment with individual RNAi clones from the ORFeome library [72] (Open Biosystems). The pilot screen consisted of plating of 2342 clones from the library; 113 either failed to growth or became contaminated, and 258 clones were not screened due to a precluding phenotype (e.g., larval arrest). Of 1971 remaining clones behaviorally screened, 173 (8.8%) were selected using low stringency visual assessment of swimming vigor; of these, eighty considered qualitatively strongest were re-screened in quadruplicate. From these re-screened clones, *raga-1* was the most reproducible across the four replicates and for this reason was selected for further analysis. Nine additional clones passed rescreening criteria in quadruplicate, but have not been further quantitatively characterized to verify the presence of enhanced swimming. Taking ten as the tentative total hits in this pilot screen, the yield was 0.5% (10/1971).

### Locomotion assays

Swimming analysis was conducted as described previously [29]. Worms were placed singly into approximately 3 ml of assay buffer (NGM liquid, without cholesterol or agar) on a standard NGM plate. At each time point over the longitudinal assay, swimming was recorded digitally in a 30s, 30 frame per second movie using StreamPix (NorPix). Offline analysis with custom programming within ImagePro (Media Cybernetics) was used to obtain spines for worms with thirteen evenly spaced points along the worm midline. Spine tracking records were reviewed for quality control, and coordinates of these points were generated and subsequently analyzed using additional custom software written in IgorPro (WaveMetrics). Cycles were reviewed to ensure the accuracy of the cycle-finding algorithm; cycles with movements out of the dorsal/ventral plane, and cycles in which animals were moving backward (which were very rare under these conditions), were excluded from analysis of swim cycle parameters. Left-right bends were identified by their appearance by review of each movie. Individual worms were analyzed for swimming parameters, then average data from individuals was pooled to produce data in age-identical cohorts.

### Transgenic worm construction

Transgenic worms bearing extra chromosomal arrays were generated with DNA at concentrations from 5–10 ng/ul using standard techniques. For tissue expression pattern and transgenic RAGA-1 mutants, expression was driven with a 4.2 kb sequence 5′ to the first gene in the predicted four-gene operon containing *raga-1* (genomic features as predicted on wormbase, www.wormbase.org, Figure S2B) as a promoter to drive eGFP or mCherry. Transgenic constructs were coinjected with either P_ofm-1_::GFP or P_myo-3_::mCherry as a marker.

### Mutant *raga-1* constructs

Mutants were generated using overlap PCR methods [73] incorporating dominant negative T18N into the *C. elegans* coding sequence (orthologous to T21N in human RagA) or gain of function Q63L (orthologous to Q66L) single residue changes. Based on prior characterization, these mutations functionally produce Rag proteins with constitutive GDP or GTP binding respectively [18], [21]. Coding sequence including intended mutations was verified by sequencing. Alignments with predicted homology were generated using VectorNTI (Invitrogen).

### CeTOR RNAi

Knockdown of CeTOR was done with a construct identical to that reported previously [37]. Adult worms were placed on RNAi plates with bacteria expressing control (empty vector) and allowed to lay eggs for several hours, then were removed. After growth at 20°C on control RNAi plates, L4 larvae were selected, and the following day were split onto control or TOR RNAi plates and placed at 25°C for lifespan assays.

### Lifespan assays

Lifespan determination was performed using animals raised at 20°C, then carried out at the temperatures indicated (20°C for lifespan assays except RNAi and dietary restriction experiments which were carried out at 25°C). Adults were scored for viability (any movement in response to prodding with a platinum pick; lack of response defined death of the animal) throughout the lifespan until death. Worms were transferred regularly to fresh plates to escape progeny and to replenish food supply. Lifespan data were analyzed using the Mantel-Cox log rank test within GraphPad Prism (GraphPad Software). Worms that left the plate, died as a result of injury or prolapsed gonad (“exploded”) or internal hatching of progeny (“bagging”) were censored at that time point following standard procedures [54], [74].

### Dietary restriction

Dietary restriction experiments were performed using the modified solid dietary restriction method as previously described [49]. Overnight cultures of OP50 *E. coli* were spun down to concentrate them and bacterial density was determined with a Petroff-Hausser counting chamber, then dilutions were made to the concentrations noted for each feeding condition. Modified NGM plates were used in dietary restriction lifespan assays, lacking peptone and containing carbenicillin (50 µg/ml) and, for the first week of the assay, FUdR (50 µg/ml) to inhibit reproduction.

### Accession number

The wormbase (www.wormbase.org) gene identification number for *raga-1* is WBGene00006414.

## Supporting Information

Figure S1 Differing rates of L/R bends in *raga-1(ok386)*, *raga-1*(GF), and wild-type N2. The mean and standard error of the number of left/right bends in each 30s movie are plotted for each genotype on the test days across the lifespan. (*) denotes significantly different from the number of bends shown by wild-type on the same day at P<.05 by Dunn's multiple comparison test.
(0.29 MB TIF)

Figure S2 Protein sequence alignments for RagA proteins and schematic diagram of the *raga-1* genomic region. (A) Predicted protein sequence alignment of *C. elegans raga-1*, human RagA, and *Drosophila melanogaster* dRagA. Note the high degree of conservation (66% and 64% with respectively with RAGA-1). (B) (Top) Schematic drawing of *raga-1* genomic region (adapted from graphics available on WormBase, www.wormbase.org). (Top) *raga-1* in the context of predicted operon (based on genome annotations at WormBase); there is a predicted SL1 splice acceptor preceding the first gene, and a predicted SL2 splice acceptor preceding each following gene in the operon. *raga-1* is the second of four predicted genes in the operon. Although *C. elegans* operons are not necessarily grouped by function, in some cases there are functional relationships among their constituents [56], and it is interesting to speculate that there may be some relationship among *raga-1*'s operon partners, particularly a putative Ras GTPase effector (RASSF2, T24F1.3) and a possible SAPS domain-containing PP2A phosphatase (C47G2.5). (Bottom) Schematic of *raga-1* deletion mutants showing approximate regions of *raga-1* gene deleted. See text for descriptions of predicted consequences for RAGA-1 protein.
(0.54 MB TIF)

Figure S3 Brood sizes of wild-type and *raga-1* mutant worms. Note the cold-dependent decrease in brood size for *raga-1(ok386)*, *raga-1(ok701)*, and the *raga-1*(DN) transgenic line compared to a much smaller change for wild-type. In addition there was a subjective appearance of a higher frequency of males in *raga-1(ok386)* and especially *raga-1(ok701)* (data not shown).
(0.22 MB TIF)

Figure S4 Comparison of aging effects on swimming for a *raga-1* rescue strain and *rsks-1(ok1255)*. (A,B) Mean cycle duration (+/−S.E.M.) on day 1 (A) and day 16 (B) of adulthood. Transgenic animals expressing *raga-1* under its own promoter in the *raga-1(ok386)* background show an increase in cycle length reflecting partial rescue, while *rsks-1(ok1255)* show relatively short cycle durations on day 16. For N2, *raga-1* rescue, *raga-1(ok386)* and *rsks-1(ok1255)* N was 16, 16, 20, and 16 on day 1 and 3, 7, 7, and 6 on day 16 respectively. (*), difference versus wild-type N2 at P<.05 using Dunn's mulitple comparison test. (C–F) Representative curvature matrices for each genotype on day 16 of adulthood. While cycles of *rsks-1(ok1255)* animals are shorter than wild-type, they also have changes suggesting uncoordinated swimming; in (E) note that tail curvature remains in the red direction much of the time. Additional twisting and head-lifting movements were also seen which suggests animals are uncoordinated at older ages.
(2.35 MB TIF)

Figure S5 Expression pattern of *raga-1* promoter fusion in adults. (A–E). mCherry expression driven by the *raga-1* operon promoter in adult animals. Anterior to the left in each frame. (A) Expression in the distal tip cell of the somatic gonad. (B) Expression in the distal tip cell and intestinal cells. (C) Expression in probable arcade cells of the head. (D,E) Expression in unidentified head neurons. Tentatively one of these cells is identified as neuron AVK. Weaker expression also was seen in the head mesodermal cell, somatic gonad sheath and spermatheca, and other unidentified head hypodermal cells.
(1.99 MB TIF)

Figure S6 Comparison of *raga-1* RNAi effects on body size and lifespan for N2 and RNAi hypersensitive *nre-1(hd20);lin-15b(hd126)* animals.
(0.35 MB TIF)

Figure S7 Lifespan curves from *raga-1(ok386)* genetic interaction experiments. (A–F) Analysis of double mutants with other aging-related genes. (E,F) represent two independent experiments testing the interaction with *rsks-1(ok1255)*. (G) RNAi lifespan data comparing the effects of *pha-4* RNAi during adulthood on wild-type N2 and *raga-1(ok386)*.
(0.98 MB TIF)

Figure S8 Lifespan curves for *raga-1(ok386)* double mutants with (A) *eat-2(ad465)* and (B) *eat-2(ad1116)*.
(0.44 MB TIF)

Table S1 Lifespan data for *raga-1* alleles and transgenic dominant negative, gain of function, and *raga-1* rescue strains. For genotype, allele names refer to *raga-1* alleles. Transgenic strains are denoted as GF (*raga-1* gain of function) or DN (*raga-1* dominant negative). (n) indicates the number of deaths scored over the total number of worms in the experiment including all censored data points. P indicates the probability result from a Mantel-Cox log rank test compared with N2 from the respective experiment.
(0.11 MB DOC)

Table S2 Brood size data. Brood sizes are shown for wild-type N2, *raga-1* deletion alleles *ok386* and *ok701*, and transgenic *raga-1* dominant negative and gain of function animals at the cultivation temperatures indicated.
(0.03 MB DOC)

Table S3 Lifespan data from RNAi experiments. RNAi treatment was started on day 1 of adulthood, except in experiments 33 and 36 in which treatment began from hatching (i.e., lifespan measured was of the treated progeny of untreated adults). Percent change in mean lifespan calculated versus the same genotype treated with control RNAi (EV, empty L4440 vector). Probability result from Mantel-Cox log rank test comparing survival curves of RNAi treated versus control RNAi treated worms from the respective experiment.
(0.07 MB DOC)

Table S4 Lifespan results for tissue-specific expression lines. P values are from Mantel-Cox survival analysis. Percent change in lifespan and statistical comparison are made versus N2 control lifespan from the same experiment. Each experiment represents testing of an independent transgenic line. Promoters used were *rgef-1* for pan-neuronal expression, *ges-1* for gut expression, *lag-2* for distal tip cell expression, *myo-3* for muscle expression, and *pie-1* for germ line.
(0.09 MB DOC)

Table S5 Lifespan results for double mutants with *raga-1(ok386)*. For single mutants, percent change in lifespan and statistical comparison are made versus N2. For double mutants, percent change in lifespan and statistical significance are versus the respective single mutant (i.e., with versus without *raga-1(ok386)* in the background). P values from Mantel-Cox log rank test against N2 for single mutants, and against single mutants for double mutants.
(0.09 MB DOC)

Table S6 Lifespan data from dietary restriction experiments. Dietary restriction begun on day 1 of adulthood. Percent change and P vaue from Mantel-Cox log rank test comparing *raga-1(ok386)* versus N2 under the same dietary condition.
(0.06 MB DOC)

Video S1 Swimming day 1 wild-type (N2) adult.
(1.15 MB AVI)

Video S2 Swimming day 16 wild-type (N2) adult.
(1.02 MB AVI)

Video S3 Out of plane, left/right bend sample in a day 6 wild-type adult.
(1.32 MB AVI)

Video S4 Swimming day 1 *raga-1(ok386)* adult.
(1.06 MB AVI)

Video S5 Swimming day 16 *raga-1(ok386)* adult.
(1.11 MB AVI)

Video S6 Swimming day 1 *raga-1*(GF) adult.
(1.11 MB AVI)

Video S7 Swimming day 6 *raga-1(GF)* adult.
(1.12 MB AVI)
