# Supplementary figures and images for: Manipulation of Behavioral Decline in Caenorhabditis elegans with the Rag GTPase raga-1
Source: PLoS Genet. 2010 May 27;6(5):e1000972. doi: 10.1371/journal.pgen.1000972 (PMC2877737; doi:10.1371/journal.pgen.1000972)

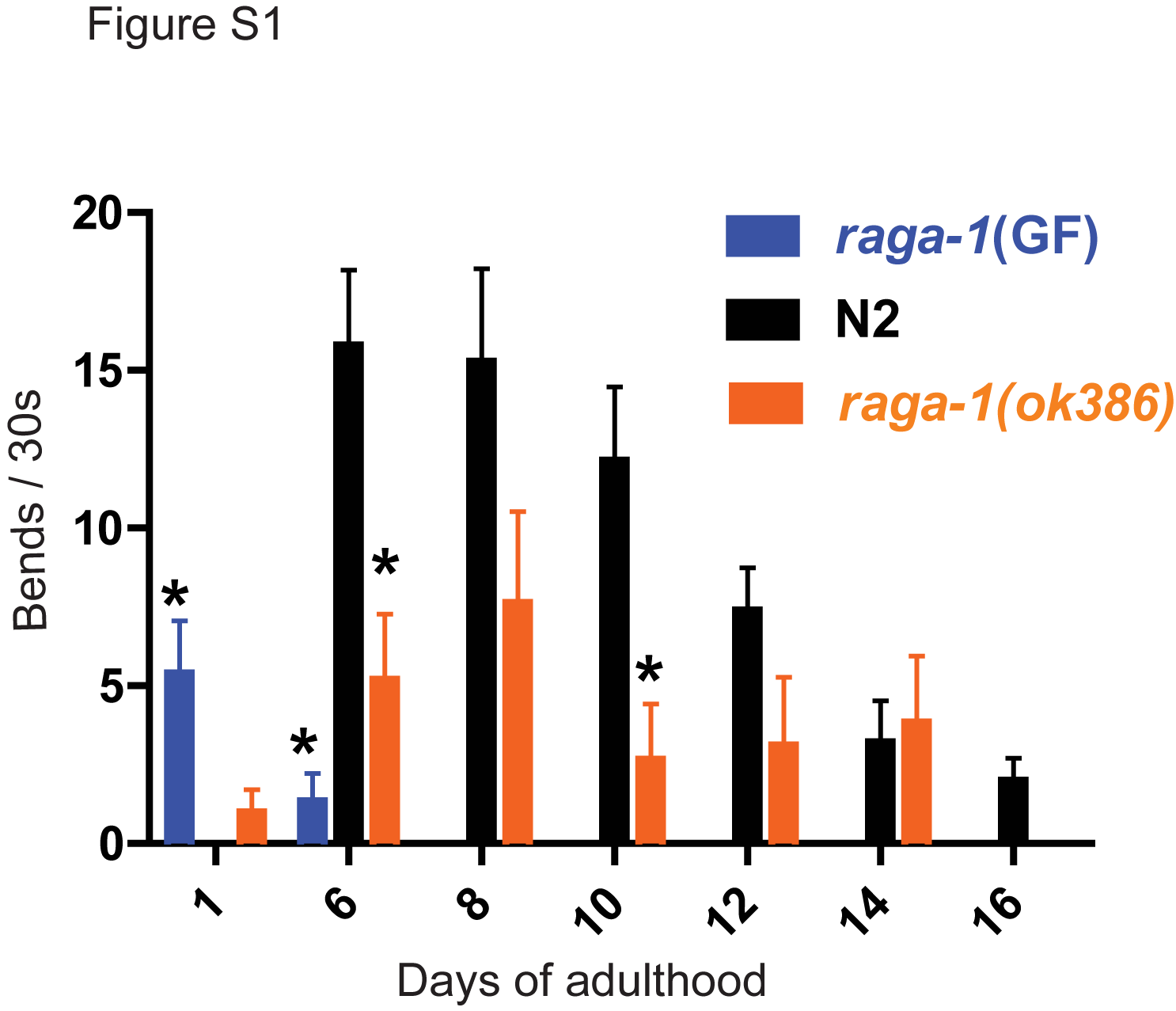

Supplement: Figure S1 — Differing rates of L/R bends in raga-1(ok386), raga-1(GF), and wild-type N2. The mean and standard error of the number of left/right bends in each 30s movie are plotted for each genotype on the test days across the lifespan. (*) denotes significantly different from the number of bends shown by wild-type on the same day at P<.05 by Dunn's multiple comparison test. (0.29 MB TIF) [file pgen.1000972.s001.tif]

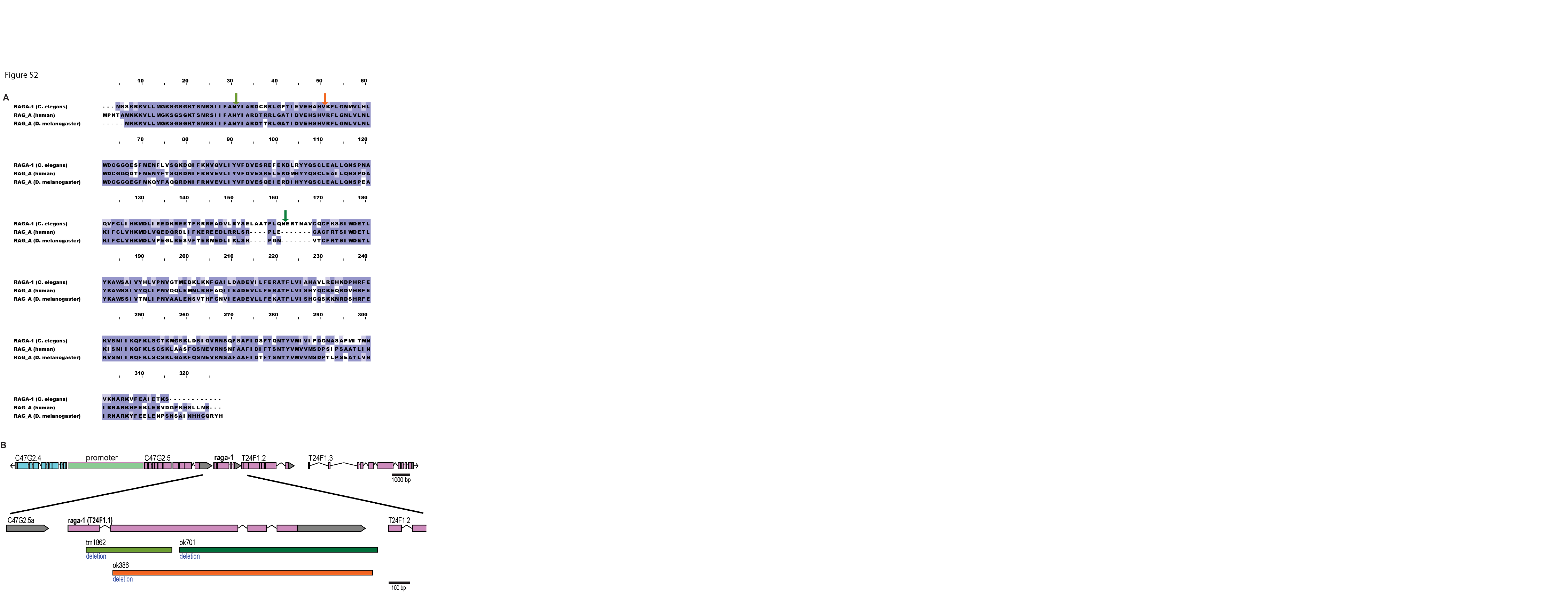

Supplement: Figure S2 — Protein sequence alignments for RagA proteins and schematic diagram of the raga-1 genomic region. (A) Predicted protein sequence alignment of C. elegans raga-1, human RagA, and Drosophila melanogaster dRagA. Note the high degree of conservation (66% and 64% with respectively with RAGA-1). (B) (Top) Schematic drawing of raga-1 genomic region (adapted from graphics available on WormBase, www.wormbase.org). (Top) raga-1 in the context of predicted operon (based on genome annotations at WormBase); there is a predicted SL1 splice acceptor preceding the first gene, and a predicted SL2 splice acceptor preceding each following gene in the operon. raga-1 is the second of four predicted genes in the operon. Although C. elegans operons are not necessarily grouped by function, in some cases there are functional relationships among their constituents [56], and it is interesting to speculate that there may be some relationship among raga-1's operon partners, particularly a putative Ras GTPase effector (RASSF2, T24F1.3) and a possible SAPS domain-containing PP2A phosphatase (C47G2.5). (Bottom) Schematic of raga-1 deletion mutants showing approximate regions of raga-1 gene deleted. See text for descriptions of predicted consequences for RAGA-1 protein. (0.54 MB TIF) [file pgen.1000972.s002.tif]

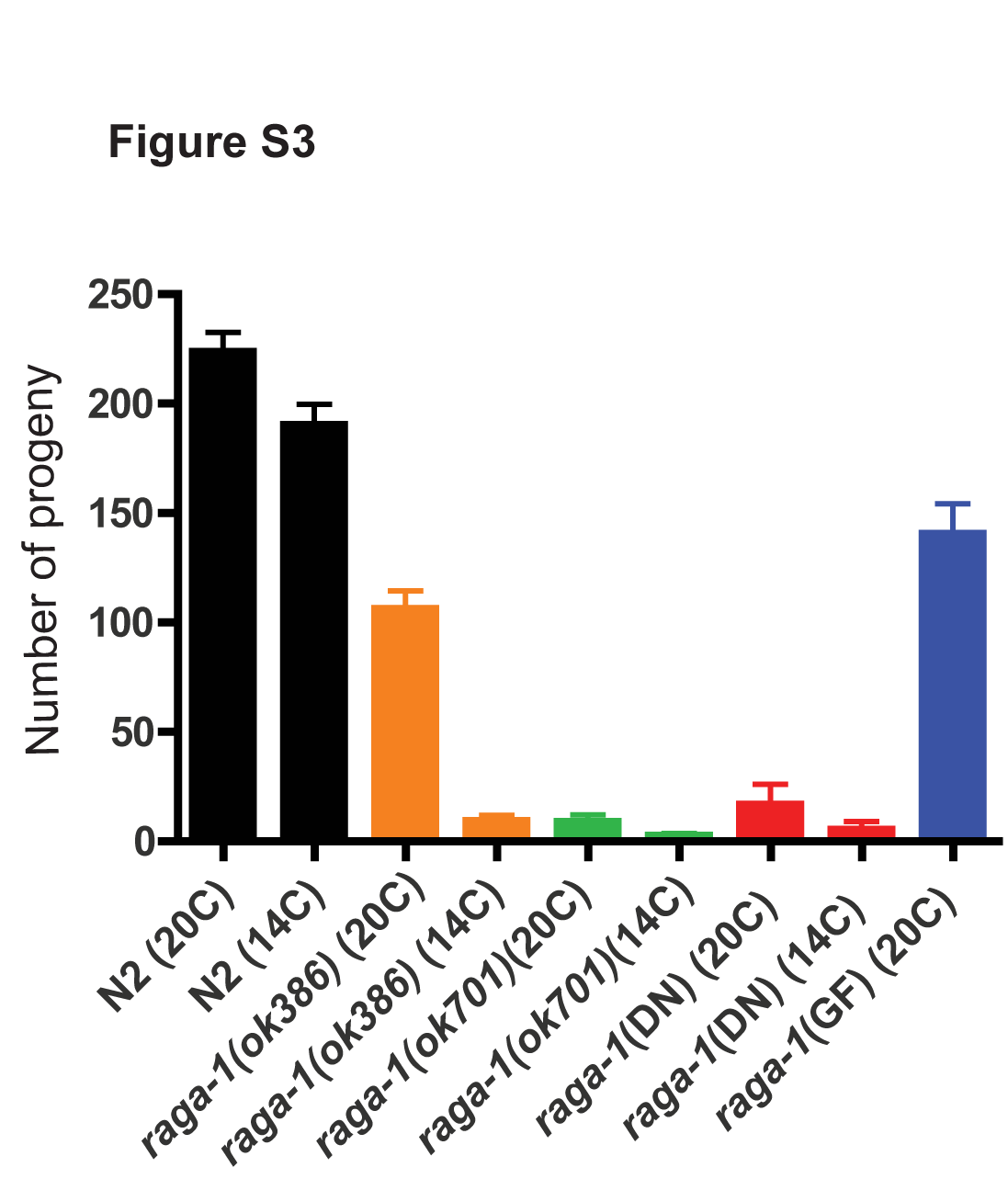

Supplement: Figure S3 — Brood sizes of wild-type and raga-1 mutant worms. Note the cold-dependent decrease in brood size for raga-1(ok386), raga-1(ok701), and the raga-1(DN) transgenic line compared to a much smaller change for wild-type. In addition there was a subjective appearance of a higher frequency of males in raga-1(ok386) and especially raga-1(ok701) (data not shown). (0.22 MB TIF) [file pgen.1000972.s003.tif]

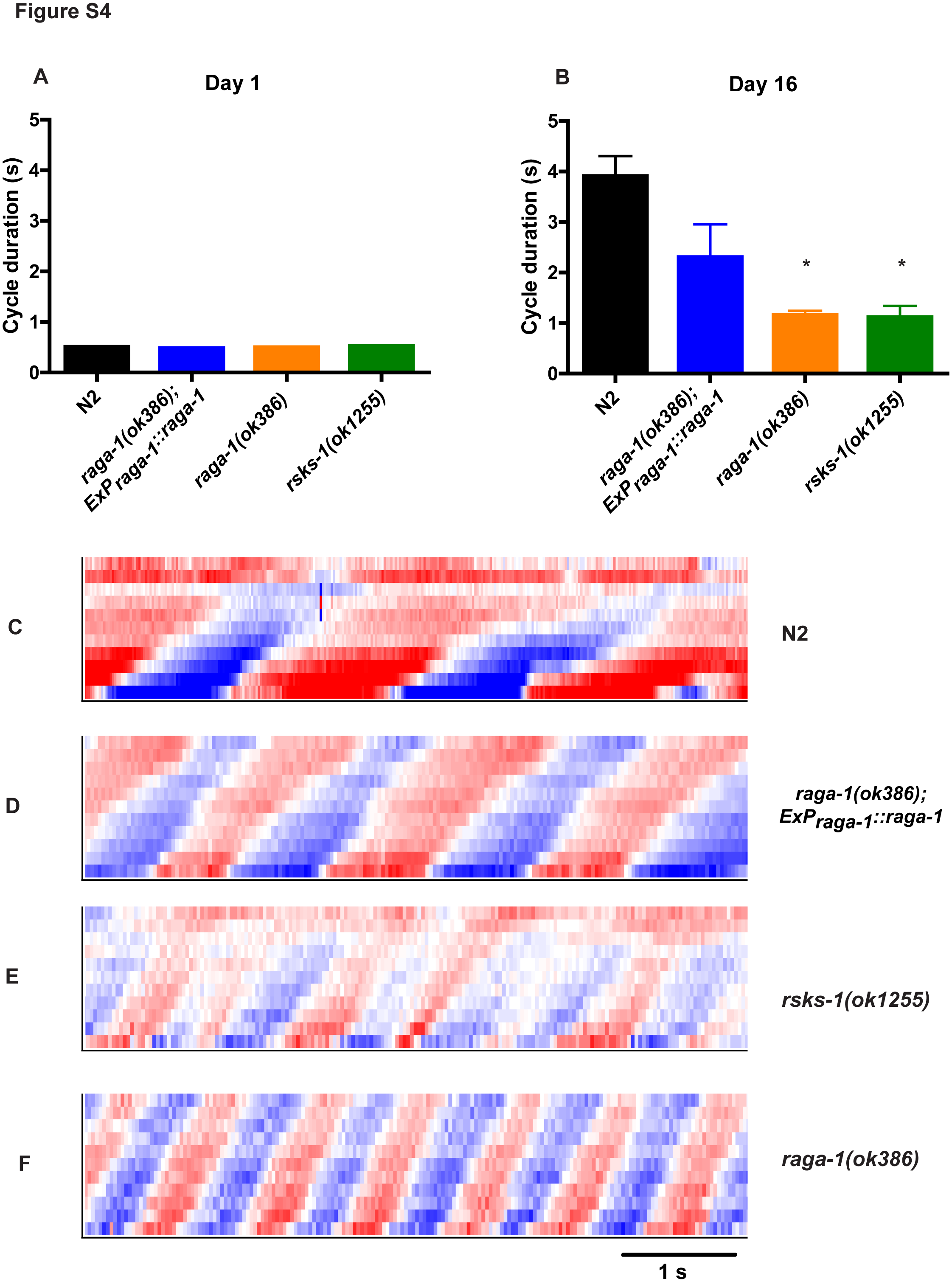

Supplement: Figure S4 — Comparison of aging effects on swimming for a raga-1 rescue strain and rsks-1(ok1255). (A,B) Mean cycle duration (+/−S.E.M.) on day 1 (A) and day 16 (B) of adulthood. Transgenic animals expressing raga-1 under its own promoter in the raga-1(ok386) background show an increase in cycle length reflecting partial rescue, while rsks-1(ok1255) show relatively short cycle durations on day 16. For N2, raga-1 rescue, raga-1(ok386) and rsks-1(ok1255) N was 16, 16, 20, and 16 on day 1 and 3, 7, 7, and 6 on day 16 respectively. (*), difference versus wild-type N2 at P<.05 using Dunn's mulitple comparison test. (C–F) Representative curvature matrices for each genotype on day 16 of adulthood. While cycles of rsks-1(ok1255) animals are shorter than wild-type, they also have changes suggesting uncoordinated swimming; in (E) note that tail curvature remains in the red direction much of the time. Additional twisting and head-lifting movements were also seen which suggests animals are uncoordinated at older ages. (2.35 MB TIF) [file pgen.1000972.s004.tif]

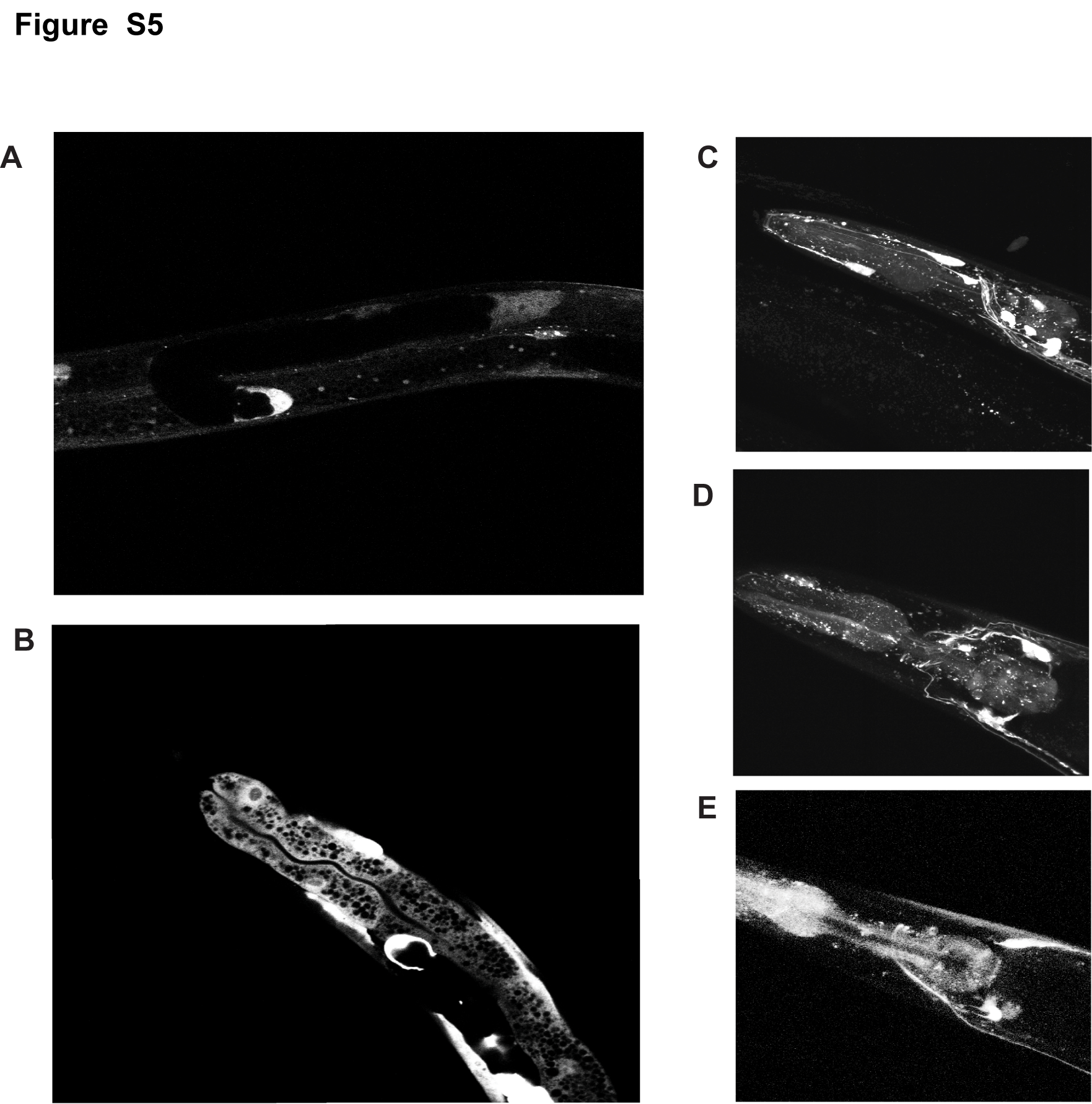

Supplement: Figure S5 — Expression pattern of raga-1 promoter fusion in adults. (A–E). mCherry expression driven by the raga-1 operon promoter in adult animals. Anterior to the left in each frame. (A) Expression in the distal tip cell of the somatic gonad. (B) Expression in the distal tip cell and intestinal cells. (C) Expression in probable arcade cells of the head. (D,E) Expression in unidentified head neurons. Tentatively one of these cells is identified as neuron AVK. Weaker expression also was seen in the head mesodermal cell, somatic gonad sheath and spermatheca, and other unidentified head hypodermal cells. (1.99 MB TIF) [file pgen.1000972.s005.tif]

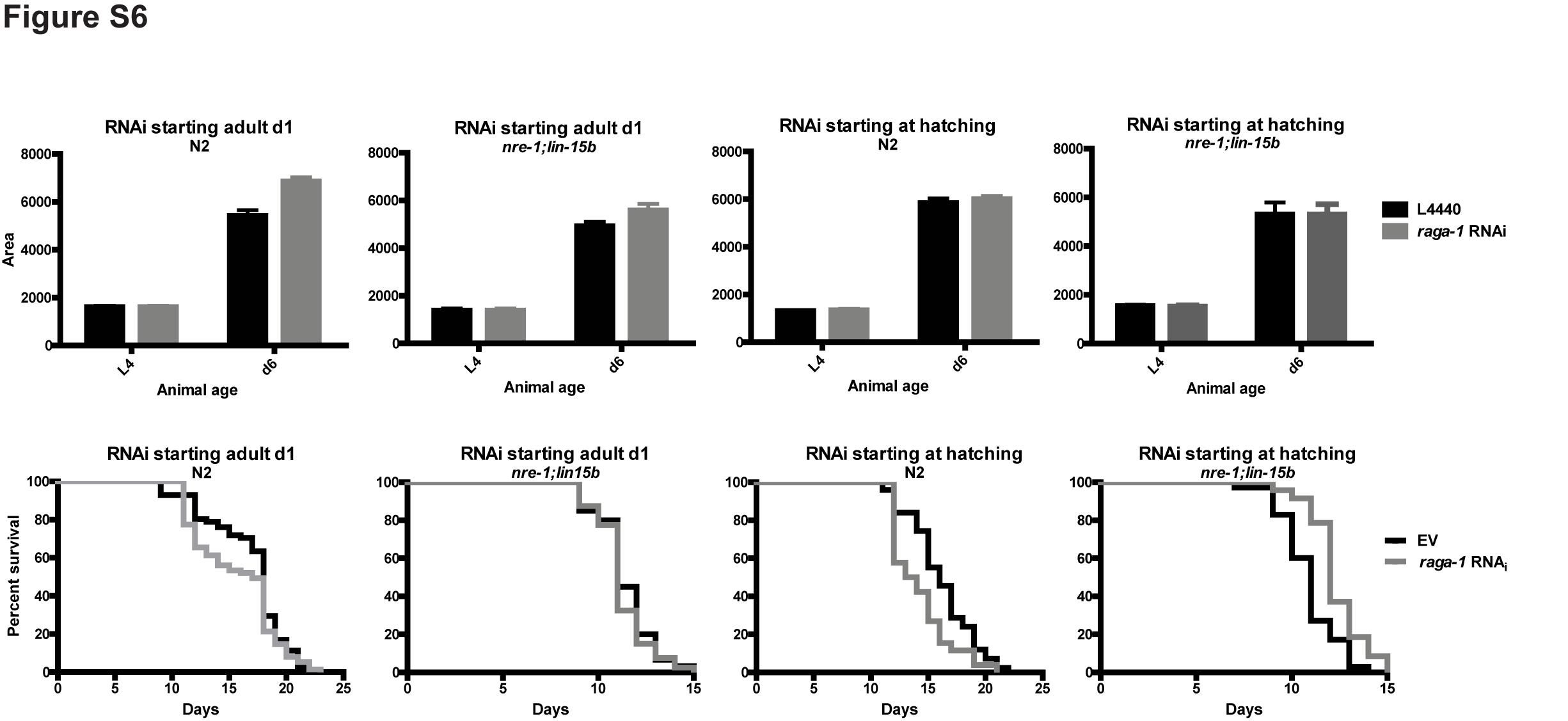

Supplement: Figure S6 — Comparison of raga-1 RNAi effects on body size and lifespan for N2 and RNAi hypersensitive nre-1(hd20);lin-15b(hd126) animals. (0.35 MB TIF) [file pgen.1000972.s006.tif]

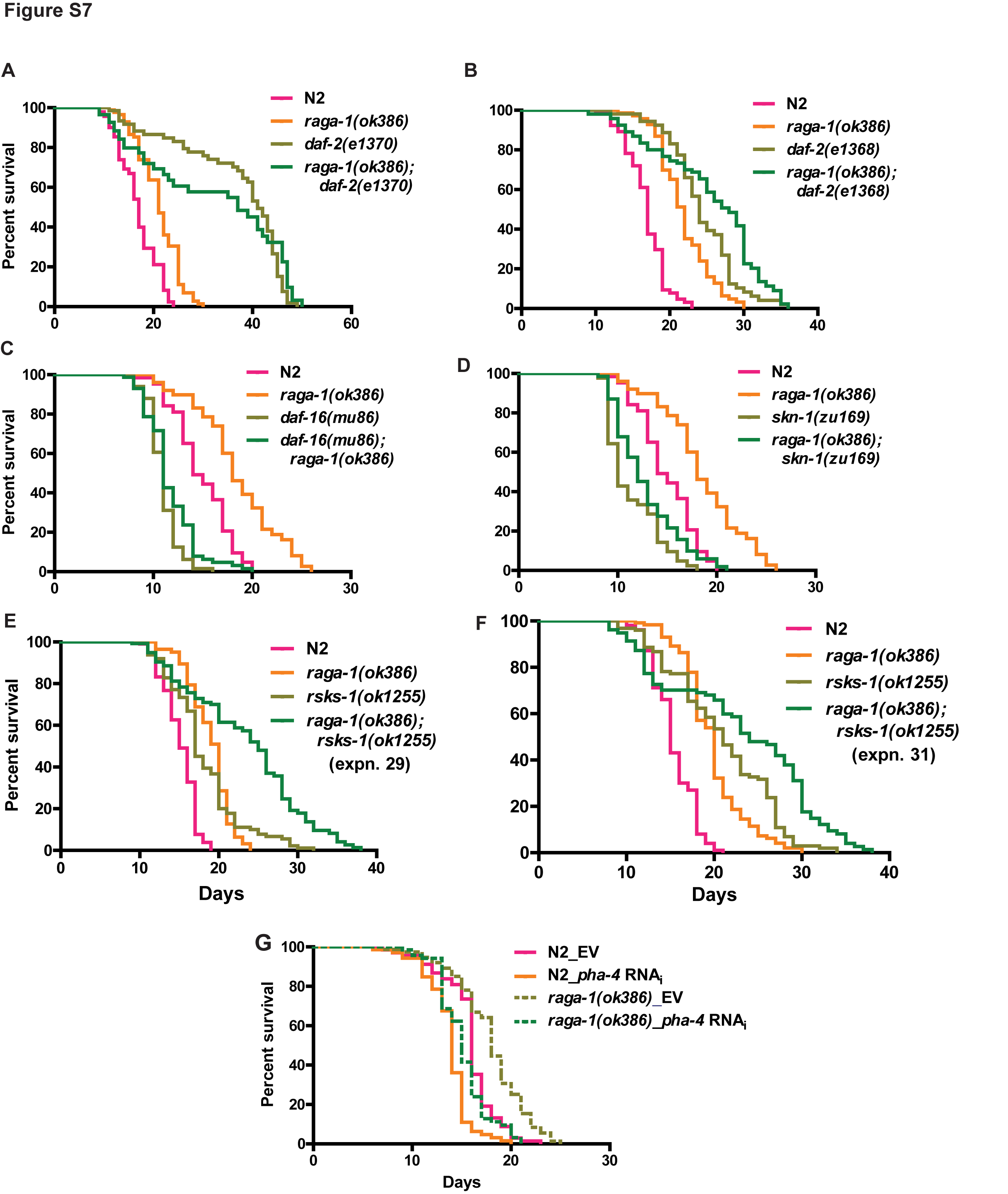

Supplement: Figure S7 — Lifespan curves from raga-1(ok386) genetic interaction experiments. (A–F) Analysis of double mutants with other aging-related genes. (E,F) represent two independent experiments testing the interaction with rsks-1(ok1255). (G) RNAi lifespan data comparing the effects of pha-4 RNAi during adulthood on wild-type N2 and raga-1(ok386). (0.98 MB TIF) [file pgen.1000972.s007.tif]

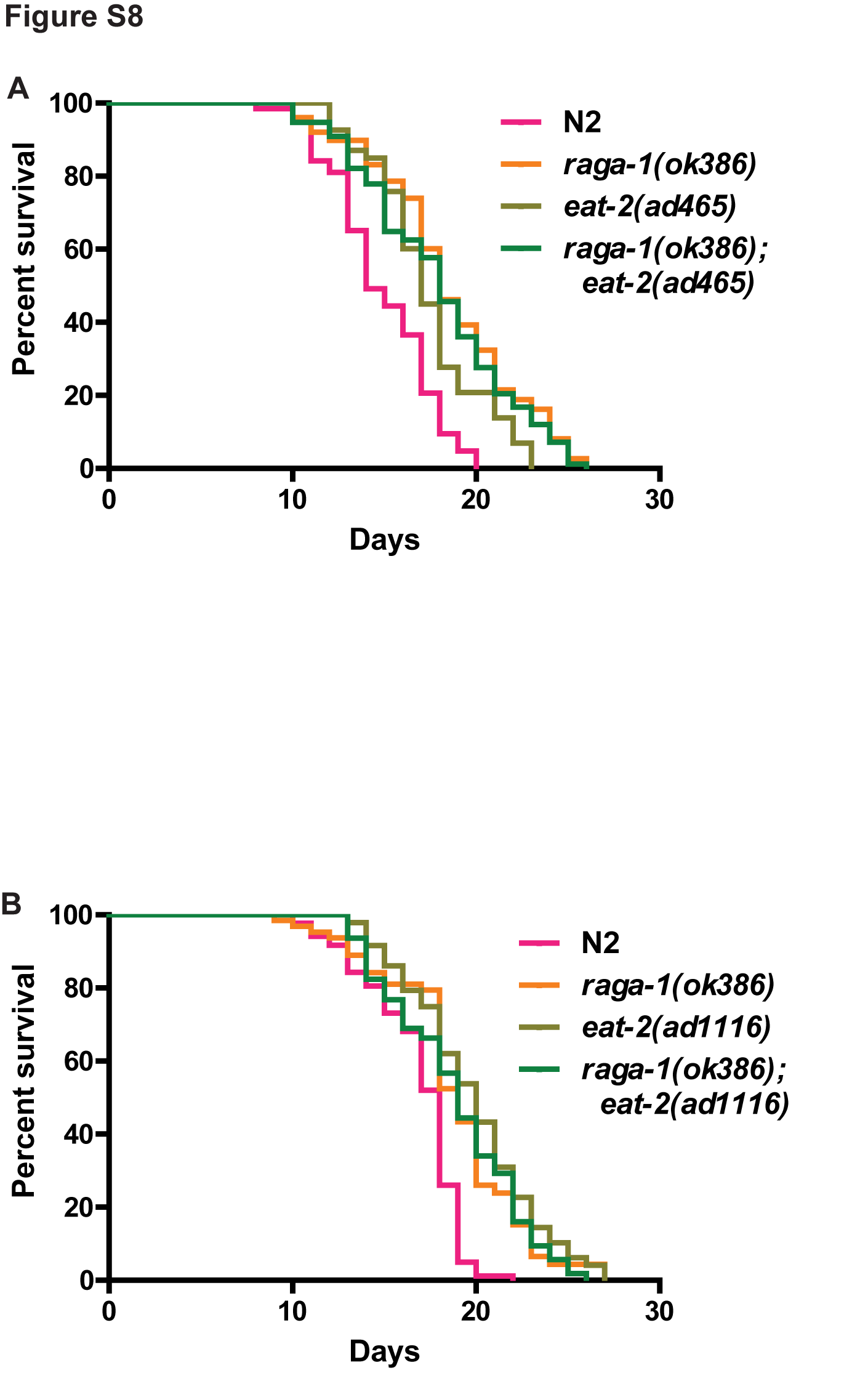

Supplement: Figure S8 — Lifespan curves for raga-1(ok386) double mutants with (A) eat-2(ad465) and (B) eat-2(ad1116). (0.44 MB TIF) [file pgen.1000972.s008.tif]
