# Supplementary material for: Manipulation of Behavioral Decline in Caenorhabditis elegans with the Rag GTPase raga-1
Source: PLoS Genet. 2010 May 27;6(5):e1000972. doi: 10.1371/journal.pgen.1000972 (PMC2877737; doi:10.1371/journal.pgen.1000972)
Supplement: Table S1 — Lifespan data for raga-1 alleles and transgenic dominant negative, gain of function, and raga-1 rescue strains. For genotype, allele names refer to raga-1 alleles. Transgenic strains are denoted as GF (raga-1 gain of function) or DN (raga-1 dominant negative). (n) indicates the number of deaths scored over the total number of worms in the experiment including all censored data points. P indicates the probability result from a Mantel-Cox log rank test compared with N2 from the respective experiment. (0.11 MB DOC) [file pgen.1000972.s009.doc]

| | Epxn | Genotype | Mean LS | (% change mean LS vs. N2) | 75%ile LS | Max. LS | n | P | | --- | --- | --- | --- | --- | --- | --- | --- | | 4 | N2 | 16.03 |  | 18 | 24 | 75/92 |  | | 4 | *ok386* | 18.8 | 17.3 | 23 | 31 | 60/88 | <.0001 | |  |  |  |  |  |  |  |  | | 6 | N2 | 14.74 |  | 17 | 22 |  |  | | 6 | *tm1862* | 14.06 | -4.6 | 16 | 30 | 224/306 | 0.424 | | 6 | DN | 18.37 | 24.6 | 22.75 | 39 | 76/109 | <.0001 | |  |  |  |  |  |  |  |  | | 7 | N2 | 15.44 |  | 18 | 24 | 48/64 |  | | 7 | *ok386* | 17.24 | 11.7 | 20 | 31 | 72/143 | .0002 | | 7 | *ok701* | 19.47 | 26.1 | 23 | 31 | 58/96 | <.0001 | |  |  |  |  |  |  |  |  | | 8 | N2 | 17.8 |  | 21 | 28 | 71/104 |  | | 8 | *ok386* | 19.84 | 11.5 | 26 | 44 | 63/86 | 0.03 | | 8 | *ok701* | 19.2 | 7.9 | 24 | 42 | 52/90 | 0.004 | | 8 | GF | 8.733 | -50.9 | 11 | 21 | 150/167 | <.0001 | |  |  |  |  |  |  |  |  | | 9 | N2 | 18.7 |  | 21 | 26 | 45/68 |  | | 9 | GF | 12.85 | -31.3 | 17 | 23 | 68/76 | <0.001 | | 9 | DN | 19.7 | 5.3 | 24 | 34 | 59/85 | 0.01 | |  |  |  |  |  |  |  |  | | 14 | N2 | 17.29 |  | 20 | 25 | 59/83 |  | | 14 | *ok386* | 23.53 | 36.1 | 26 | 46 | 56/81 | <0.001 | | 14 | DN | 19.56 | 13.1 | 22 | 29 | 45/83 | 0.0063 | | 14 | GF | 11.66 | -32.6 | 13 | 21 | 83/87 | <0.001 | |  |  |  |  |  |  |  |  | | 16 | N2 | 15.17 |  | 19 | 23 | 63/99 |  | | 16 | *ok386* | 19.22 | 26.7 | 23 | 29 | 69/111 | <0.001 | |  |  |  |  |  |  |  |  | | 17 | N2 | 16.22 |  | 18 | 22 | 58/75 |  | | 17 | *ok386* | 18.33 | 13.0 | 21 | 33 | 55/85 | .0022 | | 17 | *ok701* | 23.32 | 43.8 | 26 | 33 | 37/63 | <0.001 | | 17 | GF | 9.2 | -43.3 | 11 | 16 | 114/121 | <0.001 | | 17 | DN | 20.49 | 26.3 | 24 | 33 | 37/60 | <0.001 | |  |  |  |  |  |  |  |  | | 19 | N2 | 17.42 |  | 20 | 23 | 84/117 |  | | 19 | *ok386* | 19.67 | 12.9 | 23 | 31 | 109/160 | <.0001 | | 19 | *tm1862* | 15.19 | -12.8 | 17 | 24 | 137/162 | <.0001 | | 19 | *ok701* | 21.6 | 24.0 | 25 | 40 | 85/145 | <.0001 | | 19 | DN | 20.79 | 19.3 | 25 | 34 | 112/138 | <.0001 | |  |  |  |  |  |  |  |  | | 29 | N2 | 15.1 |  | 17 | 19 |  |  | | 29 | *ok386* | 18.7 | 23.8 | 21 | 24 | 23.8 | <.0001 | | 29 | *ok386*; P*_raga-1_*::*raga-1* | 15.1 | 0 | 17 | 19 | 0 | .31 | |  |  |  |  |  |  |  |  | | 31 | N2 | 15.3 |  | 18 | 21 | 100/164 |  | | 31 | *ok386* | 19.6 | 28.1 | 21 | 30 | 99/161 | <.0001 | | 31 | *ok386*; P*_raga-1_*::*raga-1* | 14.8 | -3.3 | 17 | 24 | 132/146 | .99 | |
| --- | --- | --- | --- | --- | --- | --- | --- | --- | --- | --- | --- | --- | --- | --- | --- | --- | --- | --- | --- | --- | --- | --- | --- | --- | --- | --- | --- | --- | --- | --- | --- | --- | --- | --- | --- | --- | --- | --- | --- | --- | --- | --- | --- | --- | --- | --- | --- | --- | --- | --- | --- | --- | --- | --- | --- | --- | --- | --- | --- | --- | --- | --- | --- | --- | --- | --- | --- | --- | --- | --- | --- | --- | --- | --- | --- | --- | --- | --- | --- | --- | --- | --- | --- | --- | --- | --- | --- | --- | --- | --- | --- | --- | --- | --- | --- | --- | --- | --- | --- | --- | --- | --- | --- | --- | --- | --- | --- | --- | --- | --- | --- | --- | --- | --- | --- | --- | --- | --- | --- | --- | --- | --- | --- | --- | --- | --- | --- | --- | --- | --- | --- | --- | --- | --- | --- | --- | --- | --- | --- | --- | --- | --- | --- | --- | --- | --- | --- | --- | --- | --- | --- | --- | --- | --- | --- | --- | --- | --- | --- | --- | --- | --- | --- | --- | --- | --- | --- | --- | --- | --- | --- | --- | --- | --- | --- | --- | --- | --- | --- | --- | --- | --- | --- | --- | --- | --- | --- | --- | --- | --- | --- | --- | --- | --- | --- | --- | --- | --- | --- | --- | --- | --- | --- | --- | --- | --- | --- | --- | --- | --- | --- | --- | --- | --- | --- | --- | --- | --- | --- | --- | --- | --- | --- | --- | --- | --- | --- | --- | --- | --- | --- | --- | --- | --- | --- | --- | --- | --- | --- | --- | --- | --- | --- | --- | --- | --- | --- | --- | --- | --- | --- | --- | --- | --- | --- | --- | --- | --- | --- | --- | --- | --- | --- | --- | --- | --- | --- | --- | --- | --- | --- | --- | --- | --- | --- | --- | --- | --- | --- | --- | --- | --- | --- | --- | --- | --- | --- | --- | --- | --- | --- | --- | --- | --- | --- | --- | --- | --- | --- | --- | --- | --- | --- | --- | --- | --- | --- | --- | --- | --- | --- | --- | --- | --- | --- | --- | --- | --- | --- | --- | --- | --- | --- | --- | --- | --- | --- | --- | --- | --- | --- | --- | --- | --- | --- | --- | --- | --- | --- | --- | --- | --- | --- | --- | --- | --- | --- | --- | --- | --- | --- | --- | --- | --- | --- | --- | --- | --- | --- | --- | --- | --- | --- | --- | --- | --- | --- | --- | --- | --- | --- | --- | --- | --- | --- | --- | --- | --- | --- | --- | --- | --- | --- | --- |
