# Supplementary material for: Manipulation of Behavioral Decline in Caenorhabditis elegans with the Rag GTPase raga-1
Source: PLoS Genet. 2010 May 27;6(5):e1000972. doi: 10.1371/journal.pgen.1000972 (PMC2877737; doi:10.1371/journal.pgen.1000972)
Supplement: Table S2 — Brood size data. Brood sizes are shown for wild-type N2, raga-1 deletion alleles ok386 and ok701, and transgenic raga-1 dominant negative and gain of function animals at the cultivation temperatures indicated. (0.03 MB DOC) [file pgen.1000972.s010.doc]

| Number of Progeny  20ºC 14ºC  Mean SEM n Mean SEM n |
| --- |
| N2 223.7 9.0 9 190.3 9.3 12 |
|  |
| *raga-1(ok386)*  106.1 8.3 12 9.2 2.8 16 |
| *raga-1(ok701)*  8.8 3.5 11 2.6 1.0 9 |
|  |
| *raga-1*(DN) 16.7 9.5 11 5.6 3.8 17 |
| *raga-1*(GF) 140.6 13.4 7 nd |
