# Supplementary material for: Manipulation of Behavioral Decline in Caenorhabditis elegans with the Rag GTPase raga-1
Source: PLoS Genet. 2010 May 27;6(5):e1000972. doi: 10.1371/journal.pgen.1000972 (PMC2877737; doi:10.1371/journal.pgen.1000972)
Supplement: Table S3 — Lifespan data from RNAi experiments. RNAi treatment was started on day 1 of adulthood, except in experiments 33 and 36 in which treatment began from hatching (i.e., lifespan measured was of the treated progeny of untreated adults). Percent change in mean lifespan calculated versus the same genotype treated with control RNAi (EV, empty L4440 vector). Probability result from Mantel-Cox log rank test comparing survival curves of RNAi treated versus control RNAi treated worms from the respective experiment. (0.07 MB DOC) [file pgen.1000972.s011.doc]

|  |  |  |  |  |  |  |  |
| --- | --- | --- | --- | --- | --- | --- | --- |
| expn. | Genotype and  RNAi treatment | mean LS | % change (mean) | 75%ile LS | Maximum  LS | n | P |
| 15 | GF_EV | 9.9 |  | 12 | 14 | 58/79 |  |
| 15 | GF_TOR | 11.8 | 19.2 | 12.75 | 18 | 56/73 | <.0001 |
| 15 | *ok386*_EV | 17.4 |  | 19 | 25 | 57/76 |  |
| 15 | *ok386*_TOR | 18.2 | 4.1 | 20 | 25 | 48/65 | 0.18 |
|  |  |  |  |  |  |  |  |
| 20 | GF_EV | 9.6 |  | 11.5 | 15 | 29/44 |  |
| 20 | GF_TOR | 12.7 | 33.3 | 15.75 | 19 | 44/54 | <.0001 |
| 20 | *ok386*_EV | 16.9 |  | 19 | 27 | 90/97 |  |
| 20 | *ok386*_TOR | 17.2 | 2.0 | 19 | 24 | 87/103 | 0.93 |
|  |  |  |  |  |  |  |  |
| 30 | N2_EV | 15.9 |  | 17 | 23 | 68/72 |  |
| 30 | N2_*pha-4* | 13.7 | -13.8 | 15 | 20 | 64/77 | <.0001 |
| 30 | *ok386*_EV | 17.9 |  | 20.75 | 25 | 72/88 |  |
| 30 | *ok386*_*pha-4* | 15.1 | -15.6 | 16 | 21 | 63/89 | <.0001 |
|  |  |  |  |  |  |  |  |
| 32 | N2_EV_adulthood only | 16.8 |  | 19 | 22 | 71/81 |  |
| 32 | N2_*raga-1*_adulthood only | 15.7 | -6.5 | 18 | 23 | 75/84 | .24 |
| 32 | *nre-1*_EV_adulthood only | 11.4 |  | 12 | 15 | 60/82 |  |
| 32 | *nre-1*_*raga-1*_adulthood only | 11.2 | -1.8 | 12 | 15 | 40/72 | .45 |
|  |  |  |  |  |  |  |  |
| 33 | N2_EV_from hatching | 16.1 |  | 18.25 | 22 | 42/75 |  |
| 33 | N2_*raga-1*_from hatching | 14.2 | -11.8 | 16 | 21 | 26/60 | .02 |
| 36 | nre-1_EV_from hatching | 10.8 |  | 12 | 14 | 70/90 |  |
| 36 | nre-1_raga-1_from hatching | 12.3 | 13.8 | 13 | 15 | 70/89 | <.0001 |
