# Supplementary material for: Manipulation of Behavioral Decline in Caenorhabditis elegans with the Rag GTPase raga-1
Source: PLoS Genet. 2010 May 27;6(5):e1000972. doi: 10.1371/journal.pgen.1000972 (PMC2877737; doi:10.1371/journal.pgen.1000972)
Supplement: Table S4 — Lifespan results for tissue-specific expression lines. P values are from Mantel-Cox survival analysis. Percent change in lifespan and statistical comparison are made versus N2 control lifespan from the same experiment. Each experiment represents testing of an independent transgenic line. Promoters used were rgef-1 for pan-neuronal expression, ges-1 for gut expression, lag-2 for distal tip cell expression, myo-3 for muscle expression, and pie-1 for germ line. (0.09 MB DOC) [file pgen.1000972.s012.doc]

| Expn. | Transgenic Genotype |  | Mean LS | Maximum  LS | N2 Control  Mean LS | % Change (vs N2) | n | P |
| --- | --- | --- | --- | --- | --- | --- | --- | --- |
| 6 | Neuronal DN | BZ1277 | 13.9 | 23 | 14.7 | -5.3 | 62/115 | .92 |
| 7 | Neuronal DN | BZ1278 | 15.5 | 25 | 15.4 | 0.5 | 55/123 | .47 |
|  |  |  |  |  |  |  |  |  |
| 6 | Gut DN | BZ1279 | 13.6 | 21 | 14.7 | -7.5 | 87/118 | .003 |
| 35 | Gut DN | BZ1280 | 15.2 | 20 | 16.7 | -8.9 | 70/105 | <.0001 |
|  |  |  |  |  |  |  |  |  |
| 6 | DTC DN | BZ1281 | 14.9 | 22 | 14.7 | 1.4 | 82/110 | .58 |
| 7 | DTC DN | BZ1282 | 16.3 | 29 | 15.4 | 5.7 | 62/94 | .10 |
| 7 | DTC DN | BZ1283 | 14.9 | 27 | 15.4 | -3.8 | 61/81 | .51 |
| 16 | DTC DN | BZ1284 | 15.0 | 23 | 15.2 | -1.0 | 41/73 | .24 |
|  |  |  |  |  |  |  |  |  |
| 29 | Neuronal GF | BZ1285 | 14.1 | 17 | 15.1 | -6.6 | 53/102 | .03 |
| 31 | Neuronal GF | BZ1286 | 13.7 | 21 | 15.3 | -10.5 | 70/124 | .02 |
|  |  |  |  |  |  |  |  |  |
| 29 | Gut GF | BZ1287 | 13.2 | 16 | 15.1 | -12.6 | 33/92 | <.0001 |
| 31 | Gut GF | BZ1288 | 14.8 | 20 | 15.3 | -8.5 | 99/188 | .39 |
|  |  |  |  |  |  |  |  |  |
| 28 | DTC GF | BZ1289 | 18.8 | 31 | 16.8 | 11.9 | 47/80 | .005 |
|  |  |  |  |  |  |  |  |  |
| 37 | Muscle DN | BZ1291 | 14.0 | 22 | 15.7 | -10.8 | 26/92 | .12 |
| 37 | Muscle DN | BZ1292 | 16.0 | 25 | 15.7 | 1.9 | 42/91 | .57 |
|  |  |  |  |  |  |  |  |  |
| 37 | Muscle GF | BZ1293 | 15.7 | 20 | 15.7 | 0 | 42/89 | .85 |
| 37 | Muscle GF | BZ1294 | 15.5 | 22 | 15.7 | -1.3 | 38/97 | .14 |
|  |  |  |  |  |  |  |  |  |
| 37 | Germ line DN | BZ1295 | 16.2 | 24 | 15.7 | 3.2 | 37/57 | .4 |
| 37 | Germ line DN | BZ1296 | 16.9 | 25 | 15.7 | 7.6 | 54/125 | .02 |
|  |  |  |  |  |  |  |  |  |
| 37 | Germ line GF | BZ1297 | 15.8 | 25 | 15.7 | 0.6 | 75/110 | .26 |
| 37 | Germ line GF | BZ1298 | 15.5 | 23 | 15.7 | -1.3 | 50/119 | .92 |
