# Supplementary material for: Manipulation of Behavioral Decline in Caenorhabditis elegans with the Rag GTPase raga-1
Source: PLoS Genet. 2010 May 27;6(5):e1000972. doi: 10.1371/journal.pgen.1000972 (PMC2877737; doi:10.1371/journal.pgen.1000972)
Supplement: Table S5 — Lifespan results for double mutants with raga-1(ok386). For single mutants, percent change in lifespan and statistical comparison are made versus N2. For double mutants, percent change in lifespan and statistical significance are versus the respective single mutant (i.e., with versus without raga-1(ok386) in the background). P values from Mantel-Cox log rank test against N2 for single mutants, and against single mutants for double mutants. (0.09 MB DOC) [file pgen.1000972.s013.doc]

|  |  |  |  |  |  |  |  |
| --- | --- | --- | --- | --- | --- | --- | --- |
| Expn. | Genotype and  RNAi treatment | Mean LS | Maximum  LS | N2 Control  Mean LS | % Change (mean LS) | n | P |
| 26 | *daf-2(e1370)* | 37.8 | 54 | 16.8 | 125.0 | 73/98 | <.0001 |
| 26 | *daf-2(e1370*); *raga-1 (ok386)* | 28.8 | 50 | 16.8 | -23.8 | 32/70 | .0062 |
| 27 | *daf-2(e1370)* | 37.2 | 59 | 16.5 | 125.5 | 69/95 | <.0001 |
| 27 | *daf-2(e1370*); *raga-1 (ok386)* | 36.7 | 55 | 16.5 | -1.3 | 36/107 | .32 |
| 28 | *daf-2(e1370)* | 36.4 | 49 | 16.8 | 116.7 | 54/99 | <.0001 |
| 28 | *daf-2(e1370*); *raga-1 (ok386)* | 29.8 | 50 | 16.8 | -18.1 | 36/151 | .88 |
|  |  |  |  |  |  |  |  |
| 26 | *daf-2(e1368)* | 24.3 | 36 | 16.8 | 44.6 | 51/75 | <.0001 |
| 26 | *daf-2(e1368*); *raga-1 (ok386)* | 25.6 | 36 | 16.8 | 5.3 | 89/122 | .0079 |
| 27 | *daf-2(e1368)* | 24.0 | 36 | 16.5 | 45.5 | 97/139 | <.0001 |
| 27 | *daf-2(e1368*); *raga-1 (ok386)* | 27.6 | 39 | 16.5 | 15.0 | 99/132 | <.0001 |
|  |  |  |  |  |  |  |  |
| 22 | *daf-16(mu86)* | 10.9 | 16 | 14.9 | -26.8 | 65/105 | <.0001 |
| 22 | *daf-16(mu86*); *raga-1 (ok386)* | 11.6 | 20 | 14.9 | 6.4 | 66/98 | .01 |
| 23 | *daf-16(mu86)* | 10.7 | 16 | 15.1 | -28.6 | 129/160 | <.0001 |
| 23 | *daf-16(mu86*); *raga-1 (ok386)* | 10.7 | 18 | 15.1 | 0 | 105/145 | .57 |
|  |  |  |  |  |  |  |  |
| 22 | *eat-2(ad465)* | 16.7 | 23 | 14.9 | 12.1 | 36/132 | <.0001 |
| 22 | *raga-1(ok386); eat-2(ad465)* | 17.6 | 26 | 14.9 | 5.3 | 88/132 | .27 |
| 23 | *eat-2(ad465)* | 13.7 | 16 | 15.1 | -8.9 | 62/121 | .26 |
| 23 | *raga-1(ok386); eat-2(ad465)* | 17.4 | 21 | 15.1 | 27.0 | 109/147 | <.0001 |
|  |  |  |  |  |  |  |  |
| 29 | *rsks-1(ok1255)* | 17.8 | 32 | 15.1 | 17.9 | 101/152 | <.0001 |
| 29 | *raga-1 (ok386); rsks-1(ok1255)* | 22.1 | 38 | 15.1 | 24.2 | 86/134 | <.0001 |
| 31 | *rsks-1(ok1255)* | 20.6 | 34 | 15.3 | 34.6 | 103/168 | <.0001 |
| 31 | *raga-1 (ok386); rsks-1(ok1255)* | 20.5 | 34 | 15.3 | 34.0 | 97/180 | .004 |
|  |  |  |  |  |  |  |  |
| 35 | *eat-2(ad1116)* | 18.9 | 27 | 16.7 | 13.2 | 71/153 | <.0001 |
| 35 | *raga-1(ok386); eat-2(ad1116)* | 18.6 | 26 | 16.7 | -1.6 | 115/168 | .08 |
|  |  |  |  |  |  |  |  |
| 22 | *skn-1(zu169)* | 11.3 | 18 | 14.9 | -24.2 | 42/59 | <.0001 |
| 22 | *skn-1(zu169);raga-1 (ok386)* | 12.6 | 21 | 14.9 | 5.3 | 55/88 | .02 |
| 23 | *skn-1(zu169)* | 10.7 | 18 | 15.1 | -28.6 | 107/140 | <.0001 |
| 23 | *skn-1(zu169);raga-1 (ok386)* | 11.8 | 24 | 15.1 | 10.3 | 58/101 | .01 |
