# Supplementary material for: Manipulation of Behavioral Decline in Caenorhabditis elegans with the Rag GTPase raga-1
Source: PLoS Genet. 2010 May 27;6(5):e1000972. doi: 10.1371/journal.pgen.1000972 (PMC2877737; doi:10.1371/journal.pgen.1000972)
Supplement: Table S6 — Lifespan data from dietary restriction experiments. Dietary restriction begun on day 1 of adulthood. Percent change and P vaue from Mantel-Cox log rank test comparing raga-1(ok386) versus N2 under the same dietary condition. (0.06 MB DOC) [file pgen.1000972.s014.doc]

| expn. | Genotype | Bacterial denisty  (cfu / ml) | mean LS | % change (mean) | 75%ile LS | Maximum  LS | n | P |
| --- | --- | --- | --- | --- | --- | --- | --- | --- |
| 24 | N2 | 5 x 10^11^ | 13.6 |  | 15 | 17 | 38/38 |  |
| 24 | *ok386* | 5 x 10^11^ | 18.4 | 35.3 | 21 | 26 | 49/49 | <.0001 |
| 24 | N2 | 5 x 10^9^ | 17.9 |  | 18.75 | 20 | 40/42 |  |
| 24 | *ok386* | 5 x 10^9^ | 21.0 | 17.3 | 23 | 27 | 53/54 | <.0001 |
| 24 | N2 | 5 x 10^7^ | 16.8 |  | 19 | 20 | 36/40 |  |
| 24 | *ok386* | 5 x 10^7^ | 18.3 | 8.9 | 19.25 | 24 | 42/47 | .008 |
|  |  |  |  |  |  |  |  |  |
| 25 | N2 | 5 x 10^11^ | 13.5 |  | 14.25 | 17 | 50/59 |  |
| 25 | *ok386* | 5 x 10^11^ | 19.8 | 46.7 | 23 | 28 | 48/60 | <.0001 |
| 25 | N2 | 5 x 10^10^ | 15.3 |  | 17 | 19 | 55/59 |  |
| 25 | *ok386* | 5 x 10^10^ | 20.7 | 35.3 | 22 | 30 | 51/60 | <.0001 |
| 25 | N2 | 5 x 10^9^ | 17.9 |  | 19 | 22 | 55/60 |  |
| 25 | *ok386* | 5 x 10^9^ | 20.4 | 14.0 | 22 | 27 | 54/58 | <.0001 |
| 25 | N2 | 5 x 10^8^ | 16.4 |  | 17 | 22 | 46/54 |  |
| 25 | *ok386* | 5 x 10^8^ | 19.3 | 17.7 | 22 | 25 | 40/44 | <.0001 |
| 25 | N2 | 5 x 10^7^ | 15.0 |  | 16 | 18 | 56/60 |  |
| 25 | *ok386* | 5 x 10^7^ | 16.6 | 10.7 | 18 | 23 | 49/55 | <.0001 |
